# The potential of point-of-care diagnostics to optimise prehospital trauma triage: a systematic review of literature

**DOI:** 10.1007/s00068-023-02226-8

**Published:** 2023-01-26

**Authors:** Leonard Stojek, Dan Bieler, Anne Neubert, Tobias Ahnert, Sebastian Imach

**Affiliations:** 1 Department of Trauma and Orthopedic Surgery, Cologne-Merheim Medical Center (CMMC), University Witten/Herdecke, Cologne, Germany; 2 Department of Orthopedics and Trauma Surgery, Reconstructive Surgery, Hand Surgery, Plastic Surgery and Burn Medicine, German Armed Forces Central Hospital Koblenz, Koblenz, Germany; 3 Department of Orthopedics and Trauma Surgery, Medical Faculty University Hospital Düsseldorf, Heinrich-Heine-University, Düsseldorf, Germany; 4 TraumaEvidence @ German Society of Traumatology, Berlin, Germany; 5 Helicopter Emergency Medical Service (HEMS) Christoph 3, Cologne, Germany

**Keywords:** Triage, Emergency medical service, Advanced trauma life support care, Focused assessment with sonography for trauma, Point-of-care testing

## Abstract

**Purpose:**

In the prehospital care of potentially seriously injured patients resource allocation adapted to injury severity (triage) is a challenging. Insufficiently specified triage algorithms lead to the unnecessary activation of a trauma team (over-triage), resulting in ineffective consumption of economic and human resources. A prehospital trauma triage algorithm must reliably identify a patient bleeding or suffering from significant brain injuries. By supplementing the prehospital triage algorithm with in-hospital established point-of-care (POC) tools the sensitivity of the prehospital triage is potentially increased. Possible POC tools are lactate measurement and sonography of the thorax, the abdomen and the vena cava, the sonographic intracranial pressure measurement and the capnometry in the spontaneously breathing patient. The aim of this review was to assess the potential and to determine diagnostic cut-off values of selected instrument-based POC tools and the integration of these findings into a modified ABCDE based triage algorithm.

**Methods:**

A systemic search on MEDLINE via PubMed, LIVIVO and Embase was performed for patients in an acute setting on the topic of preclinical use of the selected POC tools to identify critical cranial and peripheral bleeding and the recognition of cerebral trauma sequelae. For the determination of the final cut-off values the selected papers were assessed with the Newcastle–Ottawa scale for determining the risk of bias and according to various quality criteria to subsequently be classified as suitable or unsuitable. PROSPERO Registration: CRD 42022339193.

**Results:**

267 papers were identified as potentially relevant and processed in full text form. 61 papers were selected for the final evaluation, of which 13 papers were decisive for determining the cut-off values. Findings illustrate that a preclinical use of point-of-care diagnostic is possible. These adjuncts can provide additional information about the expected long-term clinical course of patients. Clinical outcomes like mortality, need of emergency surgery, intensive care unit stay etc. were taken into account and a hypothetic cut-off value for trauma team activation could be determined for each adjunct. The cut-off values are as follows: end-expiratory CO_2_: < 30 mm/hg; sonography thorax + abdomen: abnormality detected; lactate measurement: > 2 mmol/L; optic nerve diameter in sonography: > 4.7 mm.

**Discussion:**

A preliminary version of a modified triage algorithm with hypothetic cut-off values for a trauma team activation was created. However, further studies should be conducted to optimize the final cut-off values in the future. Furthermore, studies need to evaluate the practical application of the modified algorithm in terms of feasibility (e.g. duration of application, technique, etc.) and the effects of the new algorithm on over-triage. Limiting factors are the restriction with the search and the heterogeneity between the studies (e.g. varying measurement devices, techniques etc.).

**Supplementary Information:**

The online version contains supplementary material available at 10.1007/s00068-023-02226-8.

## Introduction

The accurate prehospital triage of patients who have been exposed to significant trauma mechanisms but do not show obvious signs of injury is a daily challenge in emergency services, globally. In 2012, trauma was identified as the sixth leading cause of death worldwide and is the leading cause of death and disability in patients under 35 years of age [1]. In Germany, 36,222 trauma patients were documented to be admitted to a resuscitation room and subsequently were transferred to an intensive care unit or died in the resuscitation room in 2022 [2].

Various systems have already been established in international emergency departments for triage of patients in general [3] and in particular for traumatological patients; differential criteria for the activation of trauma teams have been established [4, 5]. However, the decisive basis is always the most accurate triage possible in the prehospital setting.

The paramount requirement for optimal triage of an injured patient is to manage the allocation of medical care resources according to severity of the injury, with the greatest possible sensitivity and specificity to prevent over- or under-triage. In the case of over-triage, urgency of treatment is considered to be falsely high, often resulting in ineffective consumption of both human and economic resources [6, 7]. Under-triage is a false low estimate of urgency, which can lead to increased mortality in an injured patient due to inadequate medical care [8].

To ensure performance of triage to be as accurate as possible, various strategies have been developed in recent years [9]. Algorithm-based concepts such as "Advanced Trauma Life Support (ATLS)" use the ABCDE mnemonic, which provides the examiner with a rapid but all-embracing examination plan based on a priority list [10]. Working through a priority list is intended to give the examiner a quick but comprehensive overview of a patient's potentially life-threatening condition. Despite their intended use, triage systems often result in under-triage of 1–71.9% and over-triage of 19–79% [11]. According to the recommendations of the American College of Surgeons Committee on Trauma (ACS-COT), an under-triage rate of no more than 5% and an over-triage rate of no more than 35% should be accepted in a modern triage system [7].

In Germany, the prehospital triage of traumatological patients is determined by the "Criteria for admission to the shock room of a trauma centre by the German Society for Traumatology (DGU)" [12]. A trauma team activation is issued according to the patient's assignment to two different concepts (Grade A or Grade B) [12]. Grade A include patients with disturbance in vital parameters or recorded relevant injuries, while Grade B admits patients after assignment to a specific accident mechanism or constellation (fall from > 3 m; traffic accident ejection of an occupant or fracture of a long bone), without a necessity for the presence of obvious injuries [12, 13]. A retrospective evaluation of a level-1 trauma centre in Germany showed that increased trauma team utilisation in recent years was among other factors primarily due to the classification and assignment of patients to Grade B. However, a significant proportion of these patients classified as requiring a trauma team did not exhibit any traumatic pathologies after completion of diagnosis [14]. Consequently, there is an increased incidence of over-triage in trauma patients admitted to trauma centres according to the Grade B criteria [15]. Also considering that in 2020 29% of patients documented in the TraumaRegister DGU^®^ were 70 years and older additional triage tools seem to be necessary since those patients suffer from under-triage using non age-adapted triage criteria [16]. Altered physiological compensation mechanisms and different symptom presentation are main reasons [17, 18].

Aiming for the defined goal of an over-triage rate of less than 35% and the working groups’ hypothesis is that in-hospital already implemented point-of-care tools (POC) tools can also properly identify critically injured patients in a prehospital setting. The focus is on the earliest identification possible of injuries needing the resources of a resuscitation room i.e. critical cranial and/ or peripheral haemorrhages and the recognition of brain trauma sequelae.

The hospital-based ATLS concept defines various POC tools as so-called “adjunctions” in the primary survey helping to identify a critical injured patient. The adjuncts are capnography (at least for the intubated patient), sonography (E-FAST), X-rays of the chest and pelvis, laboratory testing including blood gas analysis (BGA) [19]. All tools were checked for the potential of prehospital use. Feasibility and existing prehospital distribution were assessment criteria.

The aim of this systematic review is to evaluate the diagnostic possibilities of the selected POC tools (lactate measurement, sonography of the abdomen, vena cava and thorax, sonographic intracranial pressure measurement and capnometry in spontaneously breathing patients) in a prehospital setting and to determine possible cut-off values for the identification of critical injuries based on defined quality criteria.

## Methods

This systematic review is reporting to PRISMA reporting guidelines [20] and as no meta-analysis was planned the plan for synthesis and the results are reported using the SWIM guidelines (Synthesis without a Meta-Analysis) [21]. The retrospectively registered study protocol can be found on PROSPERO (CRD 42022339193). After registration of the study protocol, the included study designs were formulated more precisely once. In addition, the inclusion criterion “patient age” was adjusted to any age possible and mainly adult patients cohorts (no paediatric cohorts).

### Eligibility criteria

This systematic review wants to investigate the diagnostic possibilities of the selected tools (lactate measurement, sonography of the abdomen, vena cava and thorax, sonographic intracranial pressure measurement and capnometry in spontaneously breathing patients). For this a PICOS question was formulated as shown in Table 1 below.

**Table 1:** PICO

PICO	
Patient	Patient (all ages/ no paediatric cohorts) in an acute care setting (prehospital/emergency department/ICU)
Intervention	Use of POC tools to identify critical cranial and peripheral bleeding and the recognition of cerebral trauma sequelae meaning lactate measurement, sonography of the abdomen, vena cava and thorax, sonographic intracranial pressure measurement and capnometry in spontaneously breathing patients
Comparison	Comprehensible validation of the tool
Outcome	Cut-off or measured value for specific outcome like early mortality (in-hospital mortality in first 30 days), blood transfusion or surgical emergency procedures
Study design	Randomised controlled trials and observational studies

Based on an expert consensus of emergency physicians (staff of the local HEMS) following familiarisation with the literature, the working group evaluated the following ATLS adjunctions as feasible for prehospital use: sonography independent of assessment strategy and capnometry (in spontaneously breathing patients). Instead of the BGA lactate measurement were chosen. Since BGA requires a prehospitally not established arterial puncture while lactate measurement use a similar technique like testing of blood glucose [22]. X-rays were deemed unfeasible.

Only studies published in German and English between 2000 and 2021 were eligible for inclusion. The time frame was used as the POC tools in this field are predominately evolving within the last 20 years and underwent constant technical improvement. Eligible studies shall have at least one of the specified outcomes (Table 2). Additional inclusion and exclusion criteria can be found in Table 2.

**Table 2:** Inclusion and exclusion criteria

Inclusion criteria	Exclusion criteria
Use of the sought-after instrumental diagnostic in an acute setting (prehospital, admission ED/ICU) Written in English or German Published between 2000 and 2021 Trauma cohort or partial trauma cohort, bleeding pathology Application of one of the respective tools Measurements correlating with specific outcomes (e.g. mortality, blood transfusion, surgical intervention, length of hospital stay, intracranial pressure duration, bleeding detection, etc.)	No full text available Case report, cadaver studies Study populations consisting only of children (< 18) or animals Studies with measurements after resuscitation Studies only in combination with sepsis Studies with measurements without presence of pathology Studies on training in the use of instrumental diagnostics Tool only in use with an algorithm (except sonography) Studies that determine cut-off values that indicating the change over time after several measurements “Clearance”

### Search strategy

A systemic search on MEDLINE via PubMed, LIVIVO and EMBASE for the four concepts shown in Table 3 was performed. The search was performed on 05/10/2020 and on 01.06.2022. MeSH Terms and synonyms were used and the search strategy is shown in appendix (p. 1). The search strategy was adapted to the syntax of the different databases. In addition, the bibliographies of all identified articles were screened for further suitable publications.

**Table 3:** Search terms

Search terms	
Lactate	Lactate, prehospital, point-of-care, cut-off, trauma, triage
Intracranial pressure	Sonography optic nerve, intracranial pressure, prehospital, point of care, cut-off, trauma, triage
Capnometry	Capnometry, end-tidal CO_2_, prehospital, point-of-care, cut-off, trauma, triage
Sonography	Sonography, prehospital, point-of-care, E-fast, fast, trauma, triage, vena cava

### Selection process

Further selection procedure was divided into two phases. In the first phase, only abstracts and headlines were scanned for eligibility. In the second phase, the full texts were scanned. The inclusion and exclusion criteria for both phases are listed in Table 2. No automation tools were used for the selection process and the data collection process. Upon starting phase two, the remaining studies were independently screened by two authors (LS; SI) and assessed as suitable or unsuitable. The authors of identified studies were not contacted for further clarification if the studies were deemed unclear. Disagreement or unclarity of the first two authors was solved by consulting a third author (DB).

### Data collection

Included studies were tabulated and independently assessed by both authors (LS; SI) using a non-automated standardised data extraction sheet (Word 365, Microsoft Corp., Redmond, USA). The results were then compared and recorded in a common table. If more than one outcome parameter were used, all of them were collected and included in the final evaluation. The following effect measures for each identified outcome were recorded in a table, if available: sensitivity, specificity, PPV, NPV area under curve, Odd Ratio and mean or median difference. Missing values were not calculated or inputted. Further variables that were extracted (e.g. participants, study characteristics, types of measurement etc.) can be found in the table in the appendix (pp. 17–37). The classification of the study design of the included studies was made with the Algorithm for classifying study design for questions of effectiveness [23]. The extraction sheet was evaluated after 10 studies by comparing the extraction data of LS and IS (piloting, no differences found). Disagreement of the first two authors was solved by consulting a third author (DB).

### Data synthesis, quality assessment and risk of bias

All studies that remained after the selection process were considered for the synthesis and included in the quality check. The Newcastle–Ottawa tool for assessing the risk of bias was used to score studies, with scoring based on nine domains [24]. Methodological domains/components can be found in the appendix. The assessment was performed by two authors (LS; SI), independently. Disputes were solved with discussion. To identify the final cut-off values, all cut-off values found were subjected to a quality check. This quality check consists of the following quality criteria: individual outcome, sufficient sensitivity (> 80%) or specificity (> 80%) and a Newcastle–Ottawa scale score in the upper third (7–9 points). The individual outcome is defined in detail for each tool. Depending on the diagnostic usefulness, either sensitivity or specificity is considered as a quality criterion. Sensitivity is considered for the tools for lactate measurement, intracranial pressure measurement and capnometry and specificity for sonography. Only if all quality criteria are met, the cut-off value found is considered as the final cut-off value. Tables were created to compare the outcomes of a study with the effect measures and the Newcastle–Ottawa scale, and tables were created to compare the mean and median differences in a study population for different outcome measures. A quality check table was created to provide an overview of the studies that fulfilled the criteria.

## Results

**Fig. 1:**
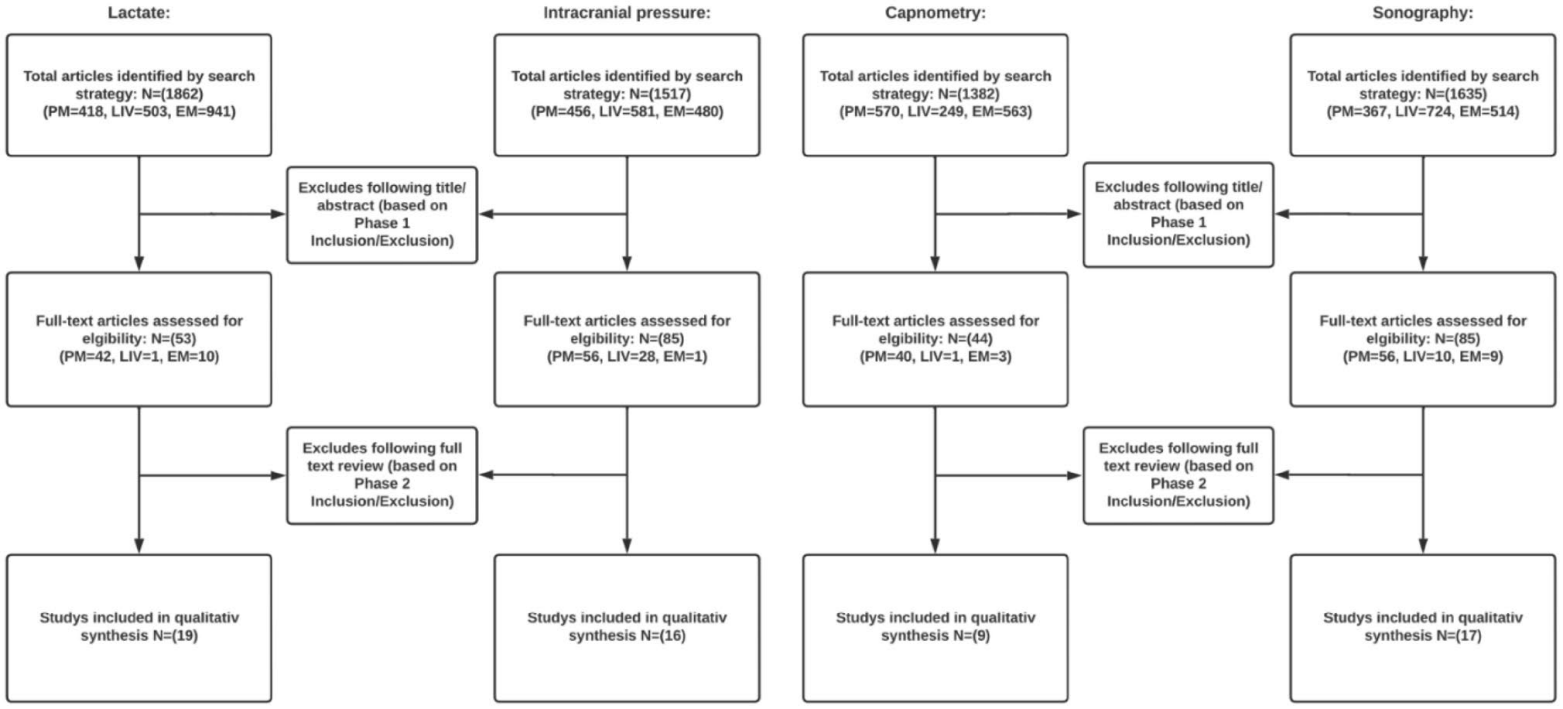
Flow chart

An overview of the selection process is given in the flowchart (Fig. 1) below. In the selection process no studies were subsequently excluded that initially appeared to meet the inclusion criteria. The most important results in relation to the correlation between the POC tools and the clinical/therapeutic courses are presented here. More detailed results including the risk of bias assessment, an overview with study population characteristics, an overview with a check of the cut-off values for the individual quality criteria as well as the final quality check can be found in tables in the appendix. Results are presented in detail for each POC tool below.

### Lactate

The search identified 1862 articles, of which 53 met the criteria for the first phase. After the second phase, 19 papers [25–43] were included in the final evaluation. The total study population in the 19 studies is 34261 participants. In 15 studies 31.1% of all participants are female and 68.9% are male [25, 26, 28–32, 34–38, 40, 41, 43]. The reported mean age from 7 studies is 40.1 years with a mean range of 31.5–62 years [26, 30, 33, 36, 40, 42, 43].

Preclinical or emergency single measure lactate levels (pLA) were correlated with resulting mortality [26, 28–32, 37, 38, 40–43] or an adjustment or need for therapy [25, 27, 29, 31, 34–36, 39, 43]. For a correlation between mortality and a lactate value, cut-off values between > 2 mmol/L (sensitivity 95%, specificity 43%, PPV 16%, NPV 99%) [43] and > 4 mmol/L (Odds ratio 1.17) [40] were determined.

Further cut-off values can be found in appendix (pp. 11–12) [28, 30–32, 37, 41]. Further cut-off values for the relationship between lactate levels and various outcomes such as need for massive transfusion, emergency surgery etc. can be found in appendix (pp. 11–12) [25, 27, 29, 33–36, 39, 43].

Regarding prehospital lactate, eight studies found significantly higher lactate levels in deceased individuals than in survivors, appendix (pp. 11–12) [26, 28–30, 37, 38, 40, 42]. The Newcastle–Ottawa scale classified four studies with nine stars [25, 29, 36, 43], six studies with eight stars [26, 28, 30, 33, 34, 39], five studies with seven stars [32, 35, 37, 38, 40], three studies with six stars [27, 31, 42] and one study with five stars [41]. The review of the cut-off values by the quality criteria identified five final cut-off values that can be assigned to either the outcome of mortality or emergent surgery. The cut-off values ranged from 2 to 3.9 mmol/l [28, 29, 36, 37, 43]. The individual outcome in the quality criteria was defined as mortality, emergent surgery and transfusion. An overview can be found in appendix (p. 16).

### Intracranial pressure

The search revealed 1517 papers, of which 85 met the criteria of the first phase. After the second phase, 16 papers [44–59] were included in the final evaluation. The total study population in the 16 studies is 1178 participants. In 12 studies, 37.1% of all participants are female and 62.8% are male [44, 45, 47, 48, 50–54, 57–59]. The reported mean age from 10 studies is 49.8 years with a mean range of 32.6–62 years [44, 45, 47, 50–52, 54, 57–59].

The correlation between optic nerve diameter (ONSD) and intracranial pressure (ICP) [45, 46, 48–59] was determined and the feasibility of the study was evaluated [44, 47].

Intracranial pressure increase (RICP) was defined either as a pressure of > 20 mmHg over a period of time [45, 47, 52, 53, 55] or clear signs of intracranial pressure on CT [46, 48–51, 54, 56–59].

The ONSD cut-off value was taken either as a single ONSD (both measured, largest value taken) [45, 55] or as the average of both eyes ONSD measurements [44, 46–54, 56–59] of a patient.

For an increase in ICP, cut-off values were determined to be between > 4.7 mm (sensitivity 100%, specificity 92%, PPV 80%, NPV 100%) [52] and 5.9 mm (sensitivity 87%, specificity 94%, PPV 93%, NPV 88%, ROC AUC 0.96) [45].

Further cut-off values for an increased ICP can be found in appendix (p.13) [46–51, 53, 54, 57–59].

In addition, four studies showed significantly higher mean ONSD values in patients with increased intracranial pressure compared to those in patients with normal intracranial pressure (appendix p. 13) [45–49, 52, 56].

A significant difference between left and right ONSD values in patients was not found [44] or a significant correlation between both eyes was found [51]. The Newcastle–Ottawa scale classified one study with nine stars [59], three studies with eight stars [50, 52, 53], four studies with six stars [45, 47, 51, 57], five studies with five stars [48, 49, 54–56] and three studies with four stars [44, 46, 58]. The review of the cut-off values by the quality criteria identified 5 final cut-off values that all fulfilled the outcome of increased intracranial pressure. The cut-off values are between 4.7 and 5.2 mm [50, 52, 59]. Increased intracranial pressure was defined as the individual outcome in the quality criteria. An overview can be found in appendix (p.16).

### Capnometry

The search identified 1382 papers, of which 44 met the criteria of the first phase. After full text screening, nine papers [60–68] were included in the final analysis. The studies investigated either intubated [60–62, 67, 68] or spontaneously breathing [63–67] patients. The total study population in the 9 studies is 2299 participants. In 8 studies, 26.3% of all participants are female and 73.6% are male [60, 62–68]. The reported mean age from 8 studies is 44.5 years with a mean range of 26.5–69 years [60, 62–68].

A correlation between preclinical end-tidal CO_2_ (ETCO_2_) and mortality [61, 62, 67, 68] as well as a correlation between end-tidal ETCO_2_ and arterial carbon dioxide partial pressure (PaCO_2_) [60] and between end-tidal ETCO_2_ and serum lactate [62, 63, 66] had been investigated.

A cut-off value for mortality in intubated patients at end-tidal ETCO_2_ was determined to be < 30 mmHg (sensitivity 89%, specificity 68%, PPV 13%, NPV 99%, ROC AUC 0.84, *p* = 0.0001) [68] or < 31 mmHg (sensitivity 93%, specificity 44%, NPV 99%) [67]. In addition, significantly decreased mean and median ETCO_2_ values were found in patients who died compared to those in survivors appendix (p. 14) [61, 62, 66–68].

Further significant differences between Mean ETCO_2_ values are found in the comparison between patients with a specific outcome (massive transfusion, necessary surgery etc.) and patients without this outcome (appendix p. 14) [63, 65, 66].

The Newcastle–Ottawa scale classified one study with nine stars [68], one study with eight stars [62], two studies with seven stars [63, 66], three studies with six stars [60, 65, 67], one study with five stars [61] and one study with four stars [64]. The review of the cut-off values through the quality criteria identifies 1 final cut-off value that meets the outcome mortality. The cut-off value is < 30 mmHg [68]. Mortality was defined as the individual outcome for the quality criteria. An overview can be found in appendix (p.16).

### Sonography

The search resulted in 1635 papers, of which 85 met the criteria of the first phase. After the second phase, 17 papers [69–85] were included. The papers were divided into the following categories: Sonography abdomen [69–71, 76–79, 83] sonography thorax [72, 73, 75, 76, 78, 79] and sonography of the vena cava [80, 81]. The total study population in the 14 studies is 7819 participants [69–82]. In nine studies, 28.2% of all participants are female and 71.9% are male [69, 71, 74, 75, 77, 79–82]. The reported mean age from eight studies is 41.1 years with a mean range of 32.6 years—63.4 years [71, 75–77, 80–82]. In abdominal ultrasonography, the detection of intraperitoneal fluid (specificity 97.5%, sensitivity 100%, PPV 94.2%, NPV 100%) and free abdominal blood (sensitivity 93%, specificity 99%, accuracy 99%) was successful. The diagnosis of hematoperitoneum was confirmed (sensitivity 46%, specificity 94.1%) [69]. The sensitivity of prehospital abdominal US for hemoperitoneum was 31.3%, specificity 96.7%, accuracy 82.1% [77]. Sensitivity and specificity of the abdominal ultrasound to detect intraperitoneal effusion were 70% and 96% while the detection of Peritoneal effusion with FAST had sensitivity 70%, specificity 96%, PPV 78%, NPV 95% and a diagnostic accuracy of 92% [79].

Emergency ultrasound findings were ruling out pneumothorax with specificity of 100% and intraabdominal fluid with a specificity of 97.1% [75].

The use of eFAST in prehospital setting had an efficiency of 95% (sensitivity 95.2%, specificity 95.2%, PPV 95.2%, NPV 95.2%) [76]. The use of POCUS to identify pneumothorax, haemothorax, and free abdominal fluid had a PPV of 100% and a NPV of 98.3% [78].

The included studies showed that ultrasound can easily detect up to 200 mL of fluid in the Morrison pouch [83]. The duration of the examinations was in 2.9 min (mean) [70, 71, 76]. Modification of prehospital management after sonography occurred in 36% [70] and in 30% [71] of patients.

Thoracic sonography showed a correct pneumothorax diagnosis in between 91,4% and 65% of all cases (sensitivity 91.4%, specificity 97%, PPV 91.4%, NPV 97%, overall accuracy 97%) [72] and (sensitivity 78%, specificity 92%, PPV 74%, NPV 94%) [73]. The detection of pneumothorax with lung sonography had sensitivity 69%, specificity 99%, PPV 94%, NPV 96% and a diagnostic accuracy of 96% [79]. The detection of Haemothorax with lung sonography had sensitivity 48%, specificity 100%, PPV 90%, NPV 97% and a diagnostic accuracy of 96% [79]. In order the highest possible values of sensitivity and specificity up to four scanning locations (anteromedial chest at the 2nd intercostal space and in the mid-clavicular line, anterolateral chest at the 4th or 5th intercostal space in the mid-axillary-line) are necessary [74]. Doing so missed pneumothoraxes were more likely to be apical and basal 7 (11.1%) vs 15 (34.9%), *p* = 0.003; 11 (17.5%) vs 18 (41.9%), *p* = 0.008, respectively). The missed pneumothoraxes were also smaller than the detected pneumothoraxes (left side: 30.7 ± 17.4 vs 12.1 ± 13.9 mm; right side: 30.2 ± 10.1 vs 6.9 ± 10.2 mm, both *p* < 0.001). If missed by eFAST, between 30 and 50% still required tube thoracostomy compared with 88.9% of those detected [84, 85].

The average (mean) application time for a thoracic ultrasound algorithm ranged from 64 s [73] to 4 min [76]. Inclusion criterion for pneumothorax was the absence of "lung sliding" and "comet-tail artefact" [72, 73, 78].

In studies measuring the diameter of the vena cava (IVC), a significant difference was detected at a blood loss of 450 mL [80] and between mean inhalation and exhalation (mean: 5.16 mm, *p* < 0.0001 and mean: 5.5 mm, *p* < 0.0001) [80].

With a blood loss of 500 mL, [81] a change in IVC after exhalation of > 1.1 mm (sensitivity 74%, specificity 77%, PPV 79.8%, NPV 70.2%) indicated previous bleeding (ROC AUC 0.79) [81]. Patients showed a significant difference in IVC after blood collection compared to before blood collection (IVCmax mean: 17.4 vs. 15.1 mm, *p* < 0.001; IVCmin mean: 10.1 vs. 8.4 mm, *p* < 0.001) [81]. These results indicate that measurement of IVC after maximal expiration is more suitable for detection of haemorrhage than measurement after maximal inspiration [81]. The Newcastle–Ottawa scale classified one study with nine stars [80], three studies with seven stars [76, 77, 81], six studies with six stars [69–73, 78] and four studies with five stars [74, 75, 79, 82]. The review of the cut-off values by the quality criteria identifies 2 studies that confirm the functionality of sonography [76, 77]. The outcome was defined as the detection of fluids or effusion in the abdomen or the detection of pneumothorax. An overview can be found in appendix (p. 16).

### Synopsis

**Fig. 2:**
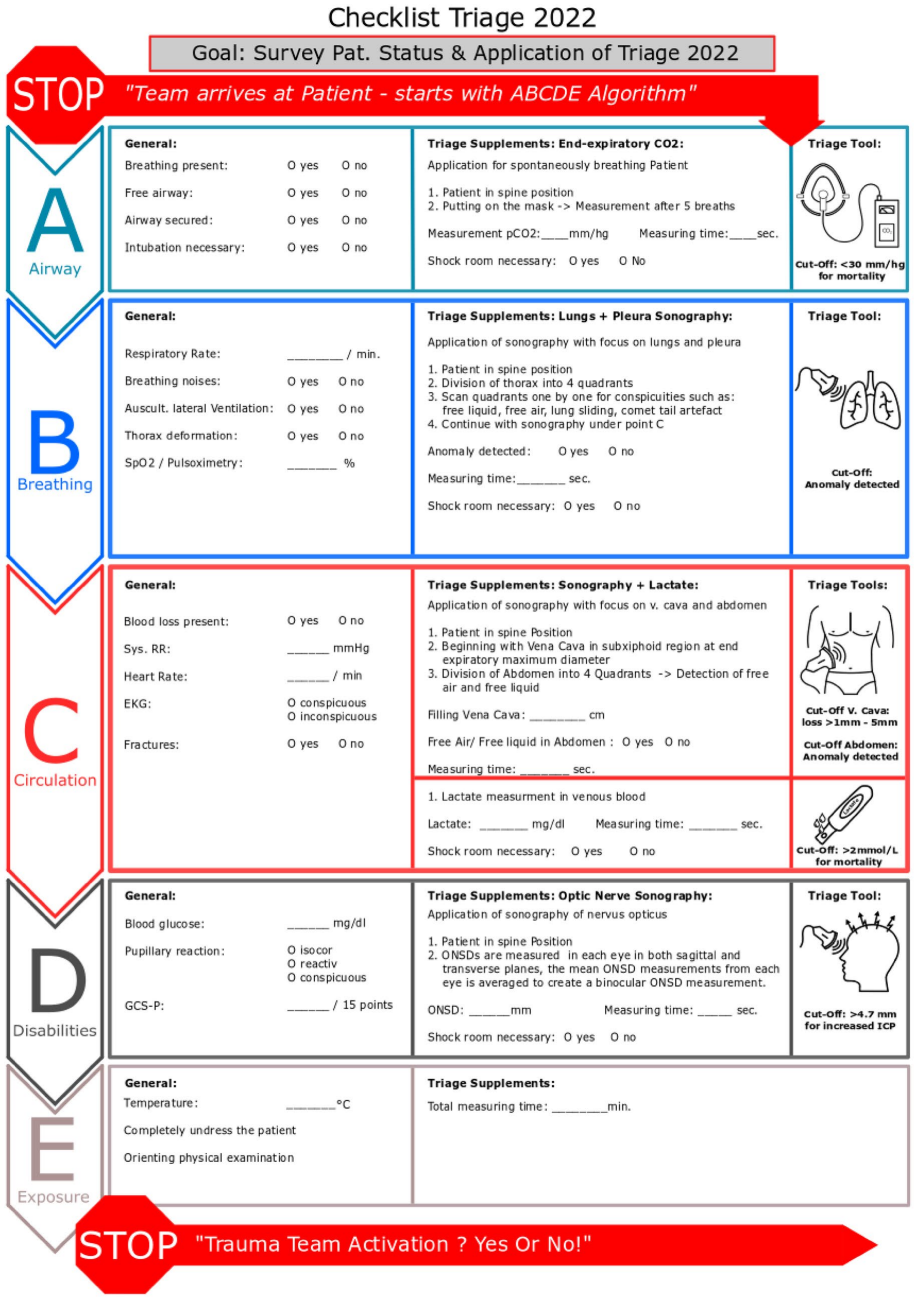
Modified algorithm

The included studies illustrate that a general prehospital application of each POC tool is, in principle, possible, and that a supplement provides additional information about the clinical status of the patient. The authors have worked out an integration of the POC tools into the ABCDE mnemonic to specify the application. The modified ABCDE mnemonic is shown in Fig. 2. In addition of the individual tools in the modified triage algorithm, the quality-tested cut-off values are also presented. It is important to note that the cut-off values can only utilised with respect to the specific outcome parameter mentioned.

This algorithm can be performed by both paramedics and emergency physicians, provided they have received the appropriate training.

Mortality within 24 h, the need for surgical intervention or transfusion are among 20 criteria defined by Waydhas and colleagues which justify the activation of a trauma team. [86] POC tools such as lactate measurement, measurement of intracranial pressure and capnometry have all been shown to be predictive of these outcome criteria. Hence, there is an indirect, hypothetical correlation with the trauma team activation. Future studies need to validate the cut-off values in terms of the trauma team activation.

Table 4 summarises the cut-off values used for the different POC diagnostics explained thereafter.

**Table 4:** Cut-off values

Point-of-care diagnostic	Cut-off value (trauma team activation)
End expiratory CO_2_	< 30 mmHg
Sonography thorax + abdomen	Anomaly detected (free liquid, free air, lung sliding etc.)
Lactate measurement	> 2 mmol/L
Optic nerve sonography	> 4.7 mm

#### A (Airway): capnometry

The detection of end-tidal CO_2_ can be done in combination with the examination of the *Airway.* The cut-off value of < 30 mmHg for mortality [68], the significantly lower mean [62, 66–68] and median [61] values in deceased compared to survivors, and the significantly lower ETCO_2_ values for a therapeutic need for surgery [63] and need for blood transfusion [65, 66] set a realistic cut-off value between > 18 and < 30 mmHg for alerting a trauma team. However, with the use of the quality criteria (shown in the appendix), only one final cut-off value in spontaneously breathing patients meets the quality requirements [68]. With < 30 mmHg, this is one of the lowest cut-off values compared to all capnometry cut-off values found, i.e. the cut-off with the lowest risk of under-triaging. However, further studies of sufficient quality need to validate this cut-off value in the future. The selected final cut-off value correlates with an increased mortality. The significant inverse relationship between serum lactate and ETCO_2_ [62, 63] can be used in combination with the lactate measurement performed under circulation as an additional diagnostic value for the prediction of the course of disease.

#### B (Breathing)—> C (circulation): sonography

The detection of a pneumothorax, an accumulation of fluid in the abdomen and the filling of the vena cava to predict blood loss can be performed in combination with the points respiration and circulation (*Breathing*). While thoracic ultrasound has started for the respiratory point, ultrasound of the abdomen and vena cava can be initiated for the circulatory point. In the quality assessment of the individual studies (shown in the appendix), special attention was paid to the specificity of the sonography. The aim was to achieve a high specificity to be able to reliably classify patients without abnormal sonographic findings as not being at risk. Although most of the included studies had a specificity of over 90% (13 out of 16) [69–73, 75–77, 79], only two studies fulfilled all quality criteria [76, 77]. The high risk of bias of these studies according to the Newcastle–Ottawa scale is the main reason. Nevertheless, two studies prove that the use of sonography is useful for the detection of abdominal fluids or effusion. For the detection of pneumothorax, no study met all quality criteria. However, four studies showed a very high sensitivity for the detection of pneumothorax, but these studies showed a high risk of bias with the Newcastle–Ottawa score (6/9) [69, 72, 73, 78]. For the measurement of the vena cava, according to Patil and colleagues the physiological diameter can be defined between 0.97 cm and 2.26 cm during exhalation and between 0.46 cm and 1.54 cm during inhalation [82].

#### C (Circulation): lactate

The use of lactate measurement in peripheral blood, with the aim of detecting bleeding can be located under *Circulation*. Reported cut-off values for mortality between 2 mmol [43] and > 4 mmol/L [40] as well as the significant differences between mean [26, 30, 40, 43] and median [28, 29, 37, 38] lactate values for deceased and survivors in eight studies suggest that a final cut-off value between 2 and 3 mmol/L can be considered optimal in relation to sensitivity and specificity. This further corroborates the findings on cut-off values for exacerbation of necessary therapeutic intervention, all of which are located above 2 mmol/L [25, 27, 29, 34–36, 38, 43]. However, the quality criteria judge that a total of five final cut-off values meet the authors' requirements [28, 29, 37, 43]. In order to minimise the risk of under-triaging, the authors decide to fix the cut-off value with the lowest threshold limit as the hypothetical final cut-off point. This is > 2 mmol/l which correlates with an increased mortality. Peripheral venous sampling, as performed in the majority of studies, is a well-established method for the determination of blood lactate levels [25–32, 35, 36, 38, 43] which requires minimal effort.

#### D (Disabilities): intracranial pressure

The detection of ICP and an associated intracranial injury is useful for the general examination of neurological symptoms in the section *Disabilities*. Cut-off values found for increased ICP were between > 4.7 mm [52] and 5.9 mm [45] of OSND. Significant differences in mean and median values between ONSD with increased and normal ICP [45–49, 52, 56] suggest a final cut-off point between 4.7 and 5.9 mm of OSND. After checking the quality criteria, five final cut-off values meet the quality requirements [50, 52, 59]. To minimise the risk of under-triage, the cut-off value with the lowest threshold limit should be used as the hypothetical final cut-off value. This is an ONSD of 4.7 mm which correlates with an increased ICP.

The "supine position" as the preferred examination position [44, 46–49, 51, 53, 54, 58, 59] could also be easily integrated into the procedure and the normally used positioning of a rescue operation. Due to the time-consuming nature of the examination, it is only recommended for GCS < 13. To combine information of “conscious level” and “brain stem” function the modified algorithm uses the GCS Pupils Score (GCS-P). The well-known GCS is combined with pupil reaction [87]. In addition to the diagnostic aspects, further studies should also evaluate any time constraints and the conditions of use. Since the time factor can contribute decisively to the outcome, especially in emergency situations, it is important to weigh up the actual benefit of additional diagnostic tests against a conventionally shorter diagnostic procedure.

Because time is the most critical factor in trauma care, it seems mandatory to define a termination time for the modified algorithm, supervised by a dedicated team member. A “stay and play” setting must be avoided by any means. Execution time of the triage algorithm is determined by the duration of the sonography part which can add up to eight minutes (FAST + OSND). By completing tasks simultaneously, a trained team should strive for a cut off value of less than five minutes. Even the feasibility of the algorithm during transport can be discussed [69].

### Limitations

The literature search and the resulting development of the modified triage algorithm is limited by several factors. First, even though, an extensive search was performed this search is limited by the restriction to only include German- and English-language studies from the years 2000 to 2021. Second, the majority of the included studies are prospective observational studies, with each observing only one of the four targeted POC tools but never in a combination of all. Similarly, the number of participants within the study populations varied relevantly, and a representative number of participants was not always achieved in the studies.

The number of selected studies is limited. The identified studies mainly investigate the diagnostic feasibility of individual tools. Furthermore, heterogeneity between studies was an important issue in the synthesis of results. Diverse conditions within the study groups, such as varying measurement devices, varying measurement techniques, diverse outcome parameters, and intubated vs. spontaneously breathing designations complicated the direct comparison and could have possibly led to a selection bias. For the modified algorithm as presented which combines all measures, the duration of application or feasibility must be examined again in a separate study.

To assess the actual functionality and causality of a modified triage algorithm, further sufficiently powered studies must be conducted in the future.

## Supplementary Information

Below is the link to the electronic supplementary material.

Supplementary file1 (DOCX 212 KB)

## Data Availability

Please contact author for data requests.
