# Supplementary material for: The potential of point-of-care diagnostics to optimise prehospital trauma triage: a systematic review of literature
Source: Eur J Trauma Emerg Surg. 2023 Jan 26;49(4):1727–39. doi: 10.1007/s00068-023-02226-8 (PMC10449679; doi:10.1007/s00068-023-02226-8)
Supplement: Supplementary file 1 — Supplementary file1 (DOCX 212 KB) [file 68_2023_2226_MOESM1_ESM.docx]

**Appendix – “Triage 2020 systematic review”**

| **Table of Content:** |  |
| --- | --- |
| 1. **Search Strategies:**………………………………………………………………………………………………………………………………………….. | **1** |
| 1. **Newcastle-Ottawa Scale:**    1. **Lactate Studies:**……………………………………………………………………………………………………………………………………….    2. **Capnometry Studies:**……………………………………………………………………………………………………………………………….    3. **Intracranial pressure Studies:**………………………………………………………………………………………………………………….    4. **Sonography Studies:**……………………………………………………………………………………………………………………………….. | **2-3**  **3**  **4**  **5** |
| 1. **Studypopulation:**     1. **Lactate Studies:**……………………………………………………………………………………………………………………………………….    2. **Intracranial Studies:**…………………………………………………………………………………………………………………………………    3. **Capnometry Studies:**……………………………………………………………………………………………………………………………….    4. **Sonography Studies:**……………………………………………………………………………………………………………………………….. | **6-7**  **7-8**  **9**  **9-10** |
| 1. **Quality Check Cut-Off:**    1. **Lactate Studies:**……………………………………………………………………………………………………………………………………….    2. **Intracranial pressure Studies:**………………………………………………………………………………………………………………….    3. **Capnometry Studies:**……………………………………………………………………………………………………………………………….    4. **Sonography Studies:**……………………………………………………………………………………………………………………………….. | **11-12**  **13**  **14**  **15** |
| 1. **Quality Check finals:**     1. **Lactate Studies:**……………………………………………………………………………………………………………………………………..    2. **Intracranial pressure Studies:**………………………………………………………………………………………………………………..    3. **Capnometry Studies:**……………………………………………………………………………………………………………………………..    4. **Sonography Studies:**……………………………………………………………………………………………………………………………... | **16**  **16**  **16**  **16** |
| 1. **Tables „Further Results“:**    1. **Lactate Studies:**……………………………………………………………………………………………………………………………………….    2. **Intracranial pressure Studies:**………………………………………………………………………………………………………………….    3. **Capnometry Studies:**……………………………………………………………………………………………………………………………….    4. **Sonography Studies:**……………………………………………………………………………………………………………………………….. | **17-21**  **22-28**  **29-31**  **32-37** |

**Search Strategies:**

**Pubmed/LIVIVO:**

**Lactate:**

(Lactate AND prehospital) OR (Lactate AND Point-of-care AND Trauma) OR (Lactate AND Point-of care AND Cut-off) OR (Lactate AND Cut-off AND Triage)

**Capnometry:**

(Capnometry AND prehospital) OR (End-tidal CO2 AND prehospital) OR (Capnometry AND trauma) OR (End-tidal CO2 AND trauma) OR (Capnometry AND Cut-off) OR (End-tidal CO2 AND Cut-off) OR (Capnometry AND triage) OR (End-tidal CO2 AND triage) OR (Capnometry AND Point-of-care) OR (End-tidal CO2 AND Point-of-care)

**Intracranial pressure:**

(Sonography AND Optic nerve AND prehospital) OR (Sonography AND Optic nerve AND Point-of-care AND Cut-off) OR (Sonography AND Optic Nerve AND Cut-off AND Triage) OR (Sonography AND Optic nerve AND prehospital AND intracranial Pressure) OR (Sonography AND Optic nerve AND prehospital AND Trauma) OR (Sonography AND Optic nerve AND Intracranial pressure)

**Sonography:**

(Sonography AND prehospital) OR (Sonography AND Point-of-care AND Trauma AND Triage) OR (E-Fast AND Sonography AND Trauma) Or (FAST AND Sonography AND Triage) OR (Sonography AND Vena Cava AND trauma) OR (Sonography AND Vena Cava AND Triage)

**EMBASE:**

**Lactate:**

(Lactate AND prehospital AND Trauma) AND ((Point of Care OR Cut off OR Triage))

**Capnometry:**

(Capnometry OR End tidal CO2) AND ((Triage AND Trauma) OR (Point of care AND Trauma) OR (Cut off AND Trauma))

**Intracranial pressure:**

(Sonography AND Optic nerve AND Trauma) AND ((intracranial pressure OR Triage OR Point of care OR Cut-off))

**Sonography:**

((Sonography OR EFAST) AND prehospital AND Trauma) AND (Triage OR Vena Cava OR Point of Care)

**Lactate Studies (Risk of bias):**

| Risk of Bias Assessment Using the Newcastle-Ottawa Scale | | | | | | | | | | | | |
| --- | --- | --- | --- | --- | --- | --- | --- | --- | --- | --- | --- | --- |
|  | **Selection** | | | |  | **Comparability** | |  | **Outcome** | | |  |
|  |  | | | |  | **Controls for** | |  |  | **Follow up** | |  |
| **Cohort** | **Representativeness of Exposed Cohort** | **Representativeness of Nonexposed Cohort** | **Ascertainment of Exposure** | **Outcome Not Previously Present** |  | **Most Important** | **Other Factors** |  | **Assessment** | **Length** | **Adequacy** | **Score** |
| Brown, JB.  2016 (25) | Yes* | Same community* | Secure record* | Yes* |  | Yes* | Yes* |  | Record linkage* | Adequate* | Adequate* | 9/9 |
| Costa L.G.  2017 (26) | Yes* | Same community * | Secure record* | Yes* |  | Yes* | No |  | Record linkage* | Adequate* | Adequate* | 8/9 |
| Fukuma, H.  2019 (27) | Yes* | Same community * | Secure record* | Yes* |  | No | No |  | Record linkage* | Adequate* | No statement | 6/9 |
| Martin Rod, F.  2020 (28) | Yes* | Same community* | Secure record* | Yes* |  | Yes* | No |  | Record linkage* | Adequate* | Adequate* | 8/9 |
| Guyette, F.  2011 (29) | Yes* | Same community* | Secure record* | Yes* |  | Yes* | Yes* |  | Record linkage* | Adequate* | Adequate* | 9/9 |
| Jansen, TC.  2008 (30) | Not representative: (17% trauma pat.) | Same community* | Secure record* | Yes* |  | Yes* | Yes* |  | Record linkage* | Adequate* | Adequate* | 8/9 |
| Van Beest, PA.  2009 (31) | Yes* | Same community* | Secure record* | No |  | No | No |  | Record linkage* | Adequate* | Adequate* | 6/9 |
| Del-Brio, P.  2020 (32) | Not representative: (8.6% trauma pat.) | Same community* | Secure record* | Yes* |  | Yes* | No |  | Record linkage* | Adequate* | Adequate* | 7/9 |
| Harrois, A.  2018 (33) | Yes* | Same community* | Secure record* | Yes* |  | Yes* | No |  | Record linkage* | Adequate* | Adequate* | 8/9 |
| Kim, Myoung J.  2021 (34) | Yes* | Same community* | Secure record* | Yes* |  | Yes* | No |  | Record linkage* | Adequate* | Adequate* | 8/9 |
| St. John, E.  2018 (35) | Yes* | Same community* | Secure record* | Yes* |  | No | No |  | Record linkage* | Adequate* | Adequate* | 7/9 |
| Guyette, F.  2015 (36) | Yes* | Same community* | Secure record* | Yes* |  | Yes* | Yes* |  | Record linkage* | Adequate* | Adequate* | 9/9 |
| Strnad, M.  2015 (37) | Yes* | Same community* | Secure record* | Yes* |  | No | No |  | Record linkage* | Adequate* | Adequate* | 7/9 |
| Baron, B.  2018 (38) | Yes* | Same community* | Secure record* | Yes* |  | No | No |  | Record linkage* | Adequate* | Adequate* | 7/9 |
| Brooke, M.  2016 (39) | Yes* | Same community* | Secure record* | Yes* |  | Yes* | No |  | Record linkage* | Adequate* | Adequate* | 8/9 |
| Gale, C.  2016 (40) | Yes* | Same community* | Secure record* | Yes* |  | No | Yes* |  | Record linkage* | Adequate* | No | 7/9 |
| Gonzalez, J.  2015 (41) | Yes* | None | Secure record* | No |  | No | No |  | Record linkage* | Adequate* | Adequate* | 5/9 |
| Kaplan, L.  2004 (42) | Yes* | Same community* | Secure record* | No |  | No | No |  | Record linkage* | Adequate* | Adequate* | 6/9 |

| Risk of Bias Assessment Using the Newcastle-Ottawa Scale | | | | | | | | | | | | |
| --- | --- | --- | --- | --- | --- | --- | --- | --- | --- | --- | --- | --- |
|  | **Selection** | | | |  | **Comparability** | |  | **Outcome** | | |  |
|  |  | | | |  | **Controls for** | |  |  | **Follow up** | |  |
| **Cohort** | **Representativeness of Exposed Cohort** | **Representativeness of Nonexposed Cohort** | **Ascertainment of Exposure** | **Outcome Not Previously Present** |  | **Most Important** | **Other Factors** |  | **Assessment** | **Length** | **Adequacy** | **Score** |
| Lavery, R.  2000 (43) | Yes* | Same community* | Secure record* | Yes* |  | Yes* | Yes* |  | Record linkage* | Adequate* | Adequate* | 9/9 |

**Capnometry Studies (Risk of bias):**

| Risk of Bias Assessment Using the Newcastle-Ottawa Scale | | | | | | | | | | | | |
| --- | --- | --- | --- | --- | --- | --- | --- | --- | --- | --- | --- | --- |
|  | **Selection** | | | |  | **Comparability** | |  | **Outcome** | | |  |
|  |  | | | |  | **Controls for** | |  |  | **Follow up** | |  |
| **Cohort** | **Representativeness of Exposed Cohort** | **Representativeness of Nonexposed Cohort** | **Ascertainment of Exposure** | **Outcome Not Previously Present** |  | **Most Important** | **Other Factors** |  | **Assessment** | **Length** | **Adequacy** | **Score** |
| Cooper, CJ.  2013 (60) | Yes* | Same community* | Secure record* | Yes* |  | No | No |  | Record linkage* | Adequate* | No | 6/9 |
| Deakin, CD  2004 (61) | Yes* | Same community* | Secure record* | No |  | No | No |  | Record linkage* | Adequate* | No | 5/9 |
| Safari, E.  2020 (62) | Yes* | Same community* | Secure record* | Yes* |  | Yes* | No |  | Record linkage* | Adequate* | Yes* | 8/9 |
| Caputo, ND.  2012 (63) | Yes* | Same community* | Secure record* | Yes* |  | No | No |  | Record linkage* | Adequate* | Yes* | 7/9 |
| Takano, Y.  2003 (64) | No | None | Secure record * | Yes* |  | No | No |  | Record linkage* | Adequate* | Unclear | 4/9 |
| Day, Darcy L.  2020 (65) | Yes* | None | Secure record* | Yes* |  | No | No |  | Record linkage* | Adequate* | Yes* | 6/9 |
| Stone, E.  2017 (66) | Yes* | Same community* | Secure record* | Yes* |  | No | No |  | Record linkage* | Adequate* | Yes* | 7/9 |
| Hunter, L.  2014 (67) | Not representative: (10% trauma pat.) | Same community* | Secure record* | Yes* |  | No | No |  | Record linkage* | Adequate* | Yes* | 6/9 |

|  | **Selection** | | | |  | **Comparability** | |  | **Outcome** | |  |
| --- | --- | --- | --- | --- | --- | --- | --- | --- | --- | --- | --- |
|  |  | | | |  | **Controls for** | |  |  |  |  |
| **Cross-sectional** | **Representativeness** | **Sample Size** | **Nonrespondents** | **Ascertainment of Exposure** |  | **Most Important** | **Other Factors** |  | **Assessment of Outcome** | **Statistical Test:** | **Score** |
| Childress K.  2018 (68) | Yes* | Justified* | No concern* | No concern** |  | Yes* | No |  | Record linkage** | Appropriate* | 9/10 |

**Intracranial pressure Studies (Risk of bias):**

| Risk of Bias Assessment Using the Newcastle-Ottawa Scale | | | | | | | | | | | | |
| --- | --- | --- | --- | --- | --- | --- | --- | --- | --- | --- | --- | --- |
|  | **Selection** | | | |  | **Comparability** | |  | **Outcome** | | |  |
|  |  | | | |  | **Controls for** | |  |  | **Follow up** | |  |
| **Cohort** | **Representativeness of Exposed Cohort** | **Representativeness of Nonexposed Cohort** | **Ascertainment of Exposure** | **Outcome Not Previously Present** |  | **Most Important** | **Other Factors** |  | **Assessment** | **Length** | **Adequacy** | **Score** |
| Houze-Cer, CH.  2019 (44) | Yes* | None | Secure records* | Yes* |  | No | No |  | Record linkage* | No | No statement | 4/9 |
| Geeraerts, T.  2007 (45) | Yes* | None | Secure records* | Yes* |  | No | No |  | Record linkage* | Adequate* | Adequate* | 6/9 |
| Blaivas, M.  2003 (46) | Yes* | None | Secure records* | Yes* |  | No | No |  | Record linkage* | No | No | 4/9 |
| Moretti, R.  2009 (47) | Yes* | None | Secure records* | Yes* |  | Yes* | No |  | Record linkage* | Adequate* | No | 6/9 |
| Goel, RS.  2008 (48) | Yes* | None | Secure records* | Yes* |  | No | No |  | Record linkage* | Adequate* | No statement | 5/9 |
| Aduayi, OS.  2015 (49) | Yes* | None | Secure records* | Yes* |  | No | No |  | Record linkage* | Adequate* | No statement | 5/9 |
| Golshani, EZ.  2015 (50) | Yes* | Same community* | Secure records* | Yes* |  | Yes* | Yes* |  | Record linkage* | Adequate* | No statement | 8/9 |
| Hanafi, MG.  2019 (51) | Yes* | None | Secure records* | Yes* |  | No | No |  | Record linkage* | Adequate* | Adequate* | 6/9 |
| Rajajee, V.  2011 (52) | Yes* | Same community* | Secure records* | Yes* |  | Yes* | Yes* |  | Record linkage* | Adequate* | No statement | 8/9 |
| Robba, C.  2020 (53) | Yes* | Same community* | Secure records* | Yes* |  | Yes* | No |  | Record linkage* | Adequate* | Adequate* | 8/9 |
| Tayal, V. S.  2007 (54) | Yes* | None | Secure records* | Yes* |  | No | No |  | Record linkage* | Adequate* | No statement | 5/9 |
| Cammarata, G.  2011 (55) | Yes* | None | Secure records* | Yes* |  | No | No |  | Record linkage* | Adequate* | No statement | 5/9 |
| Girisgin, A.S.  2007 (56) | Yes* | None | Secure records* | Yes* |  | No | No |  | Record linkage* | Adequate* | No statement | 5/9 |
| Major, Robert 2011 (57) | Yes* (50% trauma) | Same community* | Secure records* | Yes* |  | No | No |  | Record linkage* | Adequate* | No statement | 6/9 |
| Qayyum, Hasan  2013 (58) | Yes* | None | Secure records | Yes* |  | No | No |  | Record linkage* | Adequate* | No statement | 4/9 |

|  | **Selection** | | | |  | **Comparability** | |  | **Outcome** | |  |
| --- | --- | --- | --- | --- | --- | --- | --- | --- | --- | --- | --- |
|  |  | | | |  | **Controls for** | |  |  |  |  |
| **Cross-sectional** | **Representativeness** | **Sample Size** | **Nonrespondents** | **Ascertainment of Exposure** |  | **Most Important** | **Other Factors** |  | **Assessment of Outcome** | **Statistical Test** | **Score** |
| Kaur, A.  2021 (59) | Given* | Justified* | No designation | No concern** |  | Yes* | Yes* |  | Record linkage** | Appropriate* | 9/10 |

**Sonography Studies (Risk of bias):**

| Risk of Bias Assessment Using the Newcastle-Ottawa Scale | | | | | | | | | | | | |
| --- | --- | --- | --- | --- | --- | --- | --- | --- | --- | --- | --- | --- |
|  | **Selection** | | | |  | **Comparability** | |  | **Outcome** | | |  |
|  |  | | | |  | **Controls for** | |  |  | **Follow up** | |  |
| **Cohort** | **Representativeness of Exposed Cohort** | **Representativeness of Nonexposed Cohort** | **Ascertainment of Exposure** | **Outcome Not Previously Present** |  | **Most Important** | **Other Factors** |  | **Assessment** | **Length** | **Adequacy** | **Score** |
| Press, GM.  2014 (69) | Yes* | None | Secure records* | Yes* |  | None | None |  | Record linkage* | Adequate* | Adequate* | 6/9 |
| Walcher, F.  2002 (70) | Yes* | None | Secure records* | Yes* |  | None | None |  | Record linkage* | Adequate* | Adequate* | 6/9 |
| Walcher, F.  2006 (71) | Yes* | None | Secure records* | Yes* |  | None | None |  | Record linkage* | Adequate* | Adequate* | 6/9 |
| Donmez, H.  2012 (72) | Yes* | None | Secure records* | Yes* |  | None | None |  | Record linkage* | Adequate* | Adequate* | 6/9 |
| Ziapour, B.  2015 (73) | Yes* | None | Secure records* | Yes* |  | None | None |  | Record linkage* | Adequate* | Adequate* | 6/9 |
| Kirkpatrick, AW.  2004 (74) | Yes* | None | Secure records* | Yes* |  | None | None |  | Record linkage* | Adequate* | No statement | 5/9 |
| Scharonow  2018 (75) | Yes* (31% trauma) | None | Secure records* | Yes* |  | None | None |  | Record linkage* | Adequate* | No statement | 5/9 |
| Brun, PM.  2014 (76) | Yes* | Same community* | Secure records* | Yes* |  | Yes* | None |  | Record linkage* | Adequate* | No statement | 7/9 |
| Ketelaars, R.  2019 (77) | Yes* | Same community* | Secure records* | Yes* |  | None | None |  | Record linkage* | Adequate* | Adequate* | 7/9 |
| Yates, JG.  2017 (78) | Yes* | None | Secure records* | Yes* |  | None | None |  | Record linkage* | Adequate* | Adequate* | 6/9 |
| Zieleskiewicz, L.  2018 (79) | Yes* | None | Secure records* | No |  | None | None |  | Record linkage* | Adequate* | Adequate* | 5/9 |
| Lyon, M.  2005 (80) | Yes* | Same community* | Secure records* | Yes* |  | Yes* | Yes* |  | Record linkage* | Adequate* | Adequate* | 9/9 |
| Yamanoglu, A.  2019 (81) | Yes* | None | Secure records* | Yes* |  | Yes* | None |  | Record linkage* | Adequate* | Adequate* | 7/9 |
| Patil, S.  2016 (82) | Yes* | None | Secure records* | Yes* |  | None | None |  | Record linkage* | Adequate* | No statement | 5/9 |

**Lactate Studies (Studypopulation):**

| **Study:** | **Whole Study Population:** | **Female:** | **Male:** | **Age (in years):** |
| --- | --- | --- | --- | --- |
| Brown, JB.  2016 (25) | 6347 | 2018 (31.8%) | 4329 (68.2%) | Median (IQR): 44 (27-61) |
| Costa L.G.  2017 (26) | 200 | 36 (18%) | 164 (82%) | Mean (SD): 37.3 (14.63) |
| Fukuma, H.  2019 (27) | 435 in 2 Cohorts:   1. Cohort: 350 2. Cohort: 85 | 1. Cohort:   110 (31%) | 1. Cohort:   240 (69%) | 1. Cohort:   Mean: 50.5 |
|  |  | 1. Cohort:   / | 1. Cohort:   / | 1. :   / |
| Martin Rod, F.  2020 (28) | 2997 | 1242 (41.4%) | 1755 (58.6) | Median (IQR): 69 (54-81) |
| Guyette, F.  2011 (29) | 1168 | 372 (32%) | 796 (68%) | Median (IQR): 44 (27-58) |
| Jansen, TC.  2008 (30) | 124 | 51 (41%) | 73 (59%) | Mean (SD): 62 (19) |
| Van Beest, PA.  2009 (31) | 216 in 2 Groups:   1. Non shock group: 81 2. Shock group: 135 | 1. Non Shock Group:   35 (43.2%) | 1. Non Shock Group:   46 (56.8%) | 1. Non Shock Group:   Median (IQR): 54 (18-94) |
|  |  | 1. Shock Group:   54 (40%) | 1. Shock Group:   81 (60%) | 1. Shock Group:   Median (IQR): 72 (18-92) |
| Del-Brio, P.  2020 (32) | 1341 | 521 (38.9%) | 820 (61.1%) | Median (25-75th percentile):  71 (54-83) |
| Kim, Myoung J.  2021 (34) | 148 in 2 Groups:   1. Group A (arterial bleeding) (28) 2. Group B (non arterial bleeding) (120) | 1. Group A:   12 (42.9%) | 1. Group A:   16 (57.1%) | 1. Group A:   Median (IQR): 57.5 (30.5-74) |
|  |  | 1. Group B:   40 (33.3%) | 1. Group B:   80 (66.7%) | 1. Group B:   Median (IQR): 49.5 (33.0-65.0) |
| St. John, E.  2018 (35) | 314 | 86 (27.4%) | 228 (72.6) | Median (IQR): 35.5 (25-51) |
| Guyette, F.  2015 (36) | 387 in 2 Cohorts:   1. Need for RC (70) 2. No need for RC (317) | 1. Need for RC:   17 (24.5%) | 1. Need for RC:   53 (75.7%) | 1. Need for RC:   Mean (SD): 35.9 (16.5) |
|  |  | 1. No Need:   103 (32.5%) | 1. No Need:   214 (67.5%) | 1. No Need:   Mean (SD): 39.6 (17.9) |
| **Study:** | **Whole Study Population:** | **Female:** | **Male:** | **Age (in years):** |
| Strnad, M.  2015 (37) | 70 in 2 Groups:   1. Survivors (40) 2. Non-Survivors (30) | 1. Survivors:   7 (17%) | 1. Survivors:   33 (83%) | 1. Survivors:   Median (IQR): 29 (22-51) |
|  |  | 1. Non Survivors:   9 (30%) | 1. Non Survivors:   21 (70%) | 1. Non Survivors:   Median (IQR): 50 (36-72) |
| Baron, B.  2018 (38) | 10.575 | 3309 (31%) | 7266 (69%) | Median (Quartiles): 38 (25-57) |
| Brooke, M.  2016 (39) | 3468 | / | / | / |
| Gale, C.  2016 (40) | 1829 | 611 (33.4%) | 1218 (66.6%) | Mean (SD): 42.8 (18.7) |
| Gonzalez, J.  2015 (41) | 497 | 115 (23.3%) | 382 (76.7%) | Median: 45.5 |
| Kaplan, L.  2004 (42) | 282 in 2 Groups:   1. Survivors (218) 2. Non-Survivors (64) | 1. Survivors:   / | 1. Survivors:   / | 1. Survivors:   Mean (SD): 33.9 (9.4) |
|  |  | 1. Non-Survivors:   / | 1. Non-Survivors:   / | 1. Non Survivors:   Mean (SD): 31.5 (13.7) |
| Lavery, Robert  2000 (43) | 375 | 75 (20%) | 300 (80%) | Mean (SD): 35 (16.33) |
| Harrois A. 2018 (33) | 3111 | 683 (22%) | 2428 (78.0%) | Mean (SD): 38 (18) |

**Intracranial pressure Studies (Studypopulation):**

| **Study:** | **Whole Study Population:** | **Female:** | **Male:** | **Age (in years):** |
| --- | --- | --- | --- | --- |
| Houze-Cer, CH.  2019 (44) | 23 | 2 (9%) | 21 (91%) | Median (IQR): 53 (34.5-66)  Mean (SD): 51(22) |
| Geeraerts, T.  2007 (45) | 62 in 3 Groups:   1. Control (31) 2. TBI with normal ICP (16) 3. TBI with high ICP (15) | 1. Control:   7 (23%) | 1. Control:   24 (77%) | 1. Control:   Mean (SD): 55 (20) |
|  |  | 1. Normal ICP:   5 (37%) | 1. Normal ICP:   11 (63%) | 1. Normal ICP:   Mean (SD): 38 (19) |
|  |  | 1. High ICP:   4 (27%) | 1. High ICP:   11 (73%) | 1. High ICP:   Mean (SD): 38 (18) |
| Blaivas, M.  2003 (46) | 35 | / | / | / |
| Moretti, R.  2009 (47) | 106 in 3 Groups:   1. Control (53) 2. Intracranial Hemorrhage ICP < 20 (34) 3. Intracranial Hemorrhage ICP > 20 (19) | 1. Control:   20 (38%) | 1. Control:   33 (62%) | 1. Control:   Mean (SD): 58 (19) |
|  |  | 1. ICP < 20:   15 (44.2%) | 1. ICP < 20:   19 (55.8%) | 1. ICP < 20:   Mean (SD): 52 (11) |
|  |  | 1. ICP > 20:   7 (42.1%) | 1. ICP > 20:   12 (57.9) | 1. ICP > 20:   Mean (SD): 52 (12) |
| **Study:** | **Whole Study Population:** | **Female:** | **Male:** | **Age (in years):** |
| Goel, RS.  2008 (48) | 100 | 28 (28%) | 72 (72%) | Median: 28 |
| Aduayi, OS.  2015 (49) | 160 | / | / | / |
| Golshani, EZ.  2015 (50) | 131 | 30 (22.9%) | 101 (77.1%) | Mean (SD): 46.29 (10) |
| Hanafi, MG.  2019 (51) | 112 | 26 (23.2%) | 86 (76.8%) | Mean (SD): 32.59 (12.74) |
| Rajajee, V.  2011 (52) | 65 in 2 Groups:   1. With raised ICP (26) 2. Without raised ICP (39) | 1. Raised ICP:   14 (54%) | 1. Raised ICP:   12 (46%) | 1. Raised ICP:   Mean (SD): 51 (16) |
|  |  | 1. No raised ICP:   25 (64%) | 1. No raised ICP:   14 (36%) | 1. No raised ICP:   Mean (SD): 55 (16) |
| Robba, C.  2020 (53) | 100 | 45 (45%) | 55 (55%) | Median (IQR): 52 (44-62) |
| Tayal, V. S.  2007 (54) | 59 | 16 (28%) | 43 (72%) | Mean (SD): 38 (17) |
| Cammarata, G.  2011 (55) | 21 in 2 Groups:   1. Control (10) 2. Head trauma group (21) | 1. Control:   / | 1. Control:   / | 1. Control:   / |
|  |  | 1. Head trauma:   / | 1. Head trauma:   / | 1. Head trauma:   / |
| Girisgin, A.S.  2007 (56) | 54 in 2 Groups:   1. Control group (26) 2. With EICP (28) | 1. Control:   / | 1. Control:   / | 1. Control:   Mean (SD): 33.4 (16) |
|  |  | 1. With EICP:   / | 1. With EICP:   / | 1. With EICP:   / |
| Major, Robert  2011 (57) | 26 in 2 Groups:   1. Trauma (14) 2. Non-trauma (12) | 1. Trauma:   6 (42.8%) | 1. Trauma:   8 (57.2%) | 1. Trauma:   Mean: 56 |
|  |  | 1. Non-trauma:   7 (58.3%) | 1. Non-trauma:   5 (41.7%) | 1. Non-trauma:   Mean: 68 |
| Qayyum, Hasan  2013 (58) | 24 | 15 (62.5%) | 9 (37.5%) | Mean: 58 |
| Kaur, A.  2021 (59) | 100 | 19 (19%) | 81 (81%) | Mean (SD): 48.19 (14.99) |

**Capnometry Studies (Studypopulation):**

| **Study:** | **Whole Study Population:** | **Female:** | **Male:** | **Age (in years):** |
| --- | --- | --- | --- | --- |
| Childress K.  2018 (68) | 135 | 38 (28%) | 97 (72%) | Mean (SD): 40 (17) |
| Cooper, CJ.  2013 (60) | 160 | 38 (24%) | 122 (76%) | Mean (SD): 42 (19) |
| Deakin, CD  2004 (61) | 191 | / | / | Median: 31.5 |
| Safari, E.  2020 (62) | 250 | 66 (26.4%) | 184 (73.6%) | Mean (SD): 34.15 (14.77) |
| Caputo, ND.  2012 (63) | 105 | 9 (8.6%) | 96 (91.4%) | Mean: 26.5  Median (IQR): 23 (19-30) |
| Takano, Y.  2003 (64) | 41 | 11 (26.8%) | 30 (73.2%) | Mean (SD): 69 (14) |
| Day, Darcy L.  2020 (65) | 262 | 74 (28%) | 188 (72%) | Mean: 49 |
| Stone, E.  2017 (66) | 67 | 16 (24%) | 51 (76%) | Mean (SD): 41.2 (18.5) |
| Hunter, L.  2014 (67) | 1088 | 490 (45%) | 598 (55%) | Mean (SD): 54 (19) |

**Sonography Studies (Studypopulation):**

| **Study:** | **Whole Study Population:** | **Female:** | **Male:** | **Age (in years):** |
| --- | --- | --- | --- | --- |
| Press, GM.  2014 (69) | 293 | 77 (26%) | 216 (74%) | Mean (SD): 41 (17) |
| Walcher, F.  2002 (70) | 61 | / | / | / |
| Walcher, F.  2006 (71) | 230 (final Cohort: 202) | 78 (33%) | 152 (66%) | Mean (SD): 35.5 (17.9) |
| Donmez, H.  2012 (72) | 68 | / | / | / |
| Ziapour, B.  2015 (73) | 45 | / | / | / |
| Kirkpatrick, AW.  2004 (74) | 225 | 58 (26%) | 167 (74%) | Median (IQR): 37 (25-52.5) |
| Scharonow  2018 (75) | 99 | 40 (40.4%) | 59 (59.6%) | Mean (SD): 63.4 (23.7) |
| **Study:** | **Whole Study Population:** | **Female:** | **Male:** | **Age (in years):** |
| Brun, PM.  2014 (76) | 98 in 3 Groups:   1. Group 1 (44) 2. Group 2 (33) 3. Group 3 (21) | 1. Group 1:   / | 1. Group 1:   / | 1. Group 1:   Mean/median/SD: 34/28/17 |
|  |  | 1. Group 2:   / | 1. Group 2:   / | 1. Group 2:   Mean/median/SD: 39/35/29 |
|  |  | 1. Group 3:   / | 1. Group 3:   / | 1. Group 3:   Mean/median/SD: 37/35/11 |
| Ketelaars, R.  2019 (77) | 1583 | 349 (22%) | 1234 (78%) | Mean (SD): 40.5 (20.1)  Median (IQR): 39 (23-54) |
| Yates, JG.  2017 (76) | 190 | / | / | / |
| Zieleskiewicz, L.  2018 (79) | 756 | 136 (18%) | 620 (82%) | Median (25^th^,75^th^ percentile): 37 (23,49) |
| Lyon, M.  2005 (80) | 31 | 13 (41.9%) | 18 (58.1%) | Mean: 49.5 |
| Yamanoglu, A.  2019 (81) | 213 | 6 (2.8%) | 207 (97.1%) | Mean (SD): 32.6 (8.9) |
| Patil, S.  2016 (82) | 4126 | 1790 (43.3%) | 2336 (56.7%) | Mean (SD): 38.16 (5.50) |

**Lactate Studies (Cut-off):**

| **Study:** | **Cut-off: Lactate in mmol/L** | **Outcome:** | **Sensitivity: % (95% CI)** | **Specificity: %**  **(95% CI)** | **PPV: %**  **(95% CI)** | **NPV:%**  **(95% CI)** | **Area under Curve**  **(95% CI)** | **Odd Ratio**  **(95% CI)** | **Newcastle Ottawa Scale:** |
| --- | --- | --- | --- | --- | --- | --- | --- | --- | --- |
| Guyette, F. 2011 (29) | > 2 + shock criteria | Mortality | 97 (89-100) | 25 (23-28) | 7 (6-9) | 99 (98-100) | Not constructed | - | 9/9 |
| Martin Rod, F. 2020 (28) | 3.9 | Mortality | 89.6 (83.2-93.7) | 77.5 (75.9-79) | - | - | 0.876 (0.81-0.92) | 29.5 (16.8-51.7) | 8/9 |
| Jansen, TC. 2008 (30) | > 3.5: prehospital | Mortality | 75 (60-90) | 63 (53-73) | 41 (29-54) | 88 (80-96) | 0.69 (0.58-0.80) | - | 8/9 |
| Jansen, Tc. 2008 (30) | > 3.5: arrival at Ed | Mortality | 64 (47-82) | 74 (65-84) | 47 (31-63) | 85 (77-94) | 0.72 (0.60-0.84) | - | 8/9 |
| Strnad, M.  2015 (37) | > 3.4 | Mortality | 82 (63-94) | 75 (59-87) | 76% (56-90) | 81 (65-91) | 0.82 (0.70-0.03) | - | 7/9 |
| Gale, C. 2016 (40) | > 4 | Mortality | - | - | - | - | 0.6591 | 1.15 (1.08-1.22) | 7/9 |
| Van Beest, PA. 2009 (31) | 3.2 | Mortality | 75 (62-88) | 72 (62-82) | - | - | 0.775 | - | 6/9 |
| Gonzalez, J. 2015 (41) | > 4 | Mortality | - | - | - | - | Not constructed | 9.7 | 5/9 |
| Guyette, F. 2011 (29) | > 2 | In Hospital Death | - | - | - | - | Not constructed | 1.23 (1.14-1.34) | 9/9 |
| Del-Brio, P. 2020 (32) | > 2 | 2 days Mortality | - | - | - | - | 0.800 (0.74-0.85 | - | 7/9 |
| Lavery, R.  2000 (43) | > 2 VLAC | Lived/Died | 95 (84-99) | 43 (38-48) | 16 (12-21) | 99 (95-99) | Not constructed | 1.2 (1.11-1.21 | 9/9 |
| Guyette, F. 2011 (29) | > 2 + shock criteria | Emergency Operation | 86 (77-93) | 25 (22-18) | 8 (7-10) | 96 (93-98 | Not constructed | - | 9/9 |
| Guyette, F. 2011 (29) | > 2 | Emergency Operation | - | - | - | - | Not constructed | 1.13 (1.05-1.21) | 9/9 |
| Lavery, R.  2000 (43) | > 2 VLAC | Emergent Operation | 70 (59-80) | 41 (36-47) | 23 (18-29) | 85 (78-90) | Not constructed | 1.2 (1.13-1.25) | 9/9 |
| Fukuma, H. 2019 (27) | 3.1 | Massive transfusion | 64.7 | 83.5 | - | - | 0.764 (0.698-0.829) | 9.08 (8.70-9.46) | 6/9 |
| Brooke, M. 2016 (39) | > 4 | Massive transfusion | - | - | - | - | 0.71 | 10.82 (4.32-27.05) | 8/9 |
| Fukuma, H. 2019 (27) | 2.8 | Intervention of bleeding | 68.2 | 80.1 | - | - | 0.764 (0.698-0.829) | 6.82 (6.54-7.10) | 6/9 |
| Guuyette, F. 2015 (36) | > 2.5 | Resuscitative care | 93 (84-94) | 48 | - | - | 0.78 (0.73-0.83) | 3.61 (1.67-8.35) for Plac from 2.5-3,9 | 9/9 |
| St. John, E, 2018 (35) | > 2.5 | Resuscitative Care | 74.6 | 53.4 | - | - | 0.716 (0.632-0.800) | 2.27 (1.10-4.68) for Plac from 2.5-4.0 | 7/9 |

| **Study:** | **Cut-off: Lactate in mmol/L** | **Outcome:** | **Sensitivity: % (95% CI)** | **Specificity: %**  **(95% CI)** | **PPV: %**  **(95% CI)** | **NPV:%**  **(95% CI)** | **Area under Curve**  **(95% CI)** | **Odd Ratio**  **(95% CI)** | **Newcastle Ottawa Scale:** |
| --- | --- | --- | --- | --- | --- | --- | --- | --- | --- |
| Guyette, F. 2011 (29) | > 2 + shock criteria | MODS | 99 (92-100) | 25 (23-28) | 7 (6-9) | 100 (98-100) | Not constructed | - | 9/9 |
| Guyette, F. 2011 (29) | > 2 | MODS | - | - | - | - | Not constructed | 1.14 (1.03-1.23) | 9/9 |
| Lavery, R.  2000 (43) | > 2 VLAC | Admission to ICU | 81 (74-86) | 51 (45-58) | 52 (45-58) | 80 (73-87) | Not constructed | 1.5 (1.25-1.71) | 9/9 |
| Kim, Myoung J. 2021 (34) | > 3.4 | Pelvic arterial bleeding | - | - | - | - | Not constructed | 4.589 (1.354-15.552 | 8/9 |
| BROWN, JB. 2016 (25) | > 2,5 + ACS Algorithm | Trauma Center Need | 87.7 | 70 | 53.1 | 93.7 | 0.789 (0.779-0.799) | - | 9/9 |

| **Study** | **Survived in mmol/l** |  | **Deceased in mmol/l** | **P-Value** | **Newcastle Ottawa Scale** |
| --- | --- | --- | --- | --- | --- |
| Guyette, F. 2011 (29) | Median 2.3 | Vs. | Median 3.8 | < 0.0001 | 9/9 |
| Martin Rod, F. 2020 (28) | Median 2.7 |  | Median 5.7 | < 0.001 | 8/9 |
| Jansen, Tc. 2008 (30) | Mean 3.7 |  | Mean 5.3 | = 0.001 | 8/9 |
| Costa, L.G.2017 (26) | Mean 4.47 |  | Mean 6.83 | < 0.001 | 8/9 |
| Strnad, M. 2015 (37) | Median 2.6 |  | Median 5.3 | < 0.001 | 7/9 |
| Baron, B. 2018 (38) | Median 2.3 |  | Median 2.8 | = 0.0089 | 7/9 |
| Gale, C. 2016 (40) | Mean 4.20 |  | Mean 6.54 | < 0.00001 | 7/9 |
| Kaplan, L. 2004 (42) | Mean 3.6 |  | Mean 11.1 | < 0.001 | 6/9 |

**Intracranial pressure Studies (Cut-off):**

| **Study:** | **Cut-off: ONSD in mm** | **Outcome:** | **Sensitivity: % (95% CI)** | **Specificity: %**  **(95% CI)** | **PPV: %**  **(95% CI)** | **NPV: %**  **(95% CI)** | **Area under Curve**  **(95% CI)** | **Newcastle Ottawa Scale:** |
| --- | --- | --- | --- | --- | --- | --- | --- | --- |
| Kaur, A.  2021 (59) | 5.0 (mean) | Raised ICP (CT-signs) | 93.2 | 91.1 | 89.1 | 94.4 | 0.972 | 9/10 |
| Golshani, EZ.  2015 (50) | 5.0 (mean) | Raised ICP (CT-signs) | 100 (84-100) | 31.9 (23.0-41.7) | 37.3 (28.0-47.4) | 100 (85.4-100) | 0.75 (0.66-0.84) | 8/9 |
| Rajajee, V.  2011 (52) | 4.8 (single) | ICP > 20 mmHg | 96 (91-99) | 94 (92-96) | 84 (77-89) | 99 (97-100) | 0.98 (0.96-0.99) | 8/9 |
| Rajajee, V.  2011 (52) | 5.2 (single) | ICP > 25 mmHg | 98 (89-100) | 91 (88-94) | 53 (42-64) | 100 (99-100) | 0.98 (0.97-0.99) | 8/9 |
| Rajajee, V.  2011 (52) | 4.7 (mean) | ICP > 20 mmHg | 100 (85-100) | 92 (83-97) | 80 (61-92) | 100 (95-100) | 0.99 (0.95-1.00) | 8/9 |
| Robba, C.  2020 (53) | 5.3 (mean) | ICP > 20 mmHg | 70 | 75 | - | - | 0.78 (0.68-0.88) | 8/9 |
| Geeraerts, T.  2007 (45) | 5.9 (mean) | ICP > 20 mmHg | 87 | 94 | 93 | 88 | 0.96 (0.83-0.99) | 6/9 |
| Moretti, R.  2009 (47) | 5.2 (mean) | ICP >20 mmHg | 94 (88-100) | 76 (65-87) | - | - | 0.89 (0.81-0.97) | 6/9 |
| Hanafi, MG.  2019 (51) | 5.3 (mean) | Raised ICP (CT-signs) | 96.4 | 71.4 | - | - | 0.839 | 6/9 |
| Major, Robert 2011 (57) | 5.0 (mean) | Raised ICP (CT-signs) | 100 (79-100) | 86 (42-99) | 100 | 95 | Not constructed | 6/9 |
| Goel, RS.  2008 (48) | 5.0 (mean) | Raised ICP (CT-signs) | 98.6 | 92.8 | 97.26 | 96.3 | Not constructed | 5/9 |
| Aduayi, OS.  2015 (49) | 5.2 (mean) | Raised ICP (CT-signs) | 81.2 (69.9-89.6) | 100 (71.5-100) | - | - | 0.90 (0.84-0.97) | 5/9 |
| Tayal, V. S.  2007 (54) | 5.0 (mean) | Raised ICP (CT-signs) | 100 (68-100) | 63 (50-76) | 30 (12-47) | 100 (91-100) | Not constructed | 5/9 |
| Blaivas, M.  2003 (46) | 5.0 (mean) | Raised ICP (CT-signs) | 100 | 95 | 93 | 100 | Not constructed | 4/9 |
| Qayyum, Hasan  2013 (58) | 5.0 (mean) | Raised ICP (CT-signs) | 100 (83.8-100) | 75 (30.1-95.4) | 95.4 (74.13-99.75) | 100 (31-100) | Not constructed | 4/9 |

| **Study** | **Normal ICP ONSD in mm** |  | **Raised ICP ONSD in mm** | **P-Value** | **Newcastle Ottawa Scale** |
| --- | --- | --- | --- | --- | --- |
| Rajajee, V. 2011 (52) | Median 4 | Vs. | Median 5.3 | < 0.0001 | 8/9 |
| Moretti, R. 2009 (47) | Mean 5.0 |  | Mean 6.2 | < 0.01 | 6/9 |
| Geeraerts, T. 2007 (45) | Mean 5.1 |  | Mean 6.3 | < 0.0001 | 6/9 |
| Goel, RS. 2008 (48) | Mean 3.5 |  | Mean 5.8 | < 0.0001 | 5/9 |
| Aduayi, OS. 2015 (49) | Mean 4.5 |  | Mean 5.7 | = 0.0001 | 5/9 |
| Girisgin , AS. 2007 (56) | Mean 4.6 |  | Mean 6.4 | < 0.001 | 5/9 |
| Blaivas, M. 2003 (46) | Mean 4.42 |  | Mean 6.27 | = 0.001 | 4/9 |

**Capnometry Studies (Cut-off):**

| **Study:** | **Cut-off: in mmHG** | **Outcome:** | **Sensitivity: % (95% CI)** | **Specificity: %**  **(95% CI)** | **PPV: %**  **(95% CI)** | **NPV:%**  **(95% CI)** | **Area under Curve**  **(95% CI)** | **Newcastle Ottawa Scale:** |
| --- | --- | --- | --- | --- | --- | --- | --- | --- |
| Childress, K. 2018 (68) | < 30 | Mortality | 89 (51-99) | 68 (59-76) | 13 (6-24) | 99 (93-100) | 0.84 (0.67-1.00) | 9/10 |
| Hunter, Christopher L. 2014 (57) | < 31 mmHg or > 41 mmHg | Mortality | 93 (79-98) | 44 (41-48) |  | 99 (92-100) | 0.76 (0.66-0.85) | 6/9 |

| **Study** | **Outcome** | **Normal value in mmHg** |  | **Pathological value in mmHg** | **P-Value** | **Newcastle Ottawa Scale** |
| --- | --- | --- | --- | --- | --- | --- |
| Childress K. 2018 (68) | Mortality (survival vs. died) | Mean 34.0 | Vs. | Mean 18.0 | < 0.001 | 9/10 |
| Safari, E. 2020 (62) | Mortality (survival vs. died) | Mean 36.16 |  | Mean 19.59 | < 0.0001 | 8/9 |
| Stone, E. 2017 (66) | Mortality (survival vs. died) | Mean 34.6 |  | Mean 23.8 | = 0.002 | 7/9 |
| Caputo, ND. 2012 (63) | Therapeuticaly necessary surgery (no vs. yes) | Mean 29.4 |  | Mean 25.2 | = 0.006 | 7/9 |
| Stone, E. 2017 (66) | Need for massive transfusion (no vs. yes) | Mean 35.5 |  | Mean 27.5 | = 0.028 | 7/9 |
| Hunter, L. 2014 (57) | Mortality (survival vs. died) | Mean 34.0 |  | Mean 25.0 | < 0.001 | 6/9 |
| Day, Darcy L. 2020 (65) | Required blood transfusion (no vs. yes) | Mean 30.0 |  | Mean 26.0 | = 0.03 | 6/9 |
| Deakin, CD. 2004 (61) | Mortality (survival vs. died) | Median 30.75 |  | Median 26.25 | < 0.0001 | 5/9 |

**Sonography (Cut-off):**

| **Study:** | **Location of Sonography** | **Outcome:** | **Sensitivity: % (95% CI)** | **Specificity: %**  **(95% CI)** | **PPV: %**  **(95% CI)** | **NPV: %**  **(95% CI)** | **Accuracy:** | **Newcastle Ottawa Scale:** |
| --- | --- | --- | --- | --- | --- | --- | --- | --- |
| Ketelaars, R. 2019 (77) | Abdominal | Hemoperitoneum | 31.3 | 96.7 | 72.9 | 83.0 | 82.1 | 7/9 |
| Walcher, F. 2002 (70) | Abdominal | Detection intraperitoneal fluid | 100 | 97.5 | 94.2 | 100 | - | 6/9 |
| Walcher F. 2006 (71) | Abdominal | Free abdominal blood | 93 (76-99) | 99 (97-100) | - | - | 99% (96-100) | 6/9 |
| Press, GM. 2014 (69) | Abdominal | Hemoperitoneum | 46 (27.1-94.1) | 94.1 (89.2-97) | 54.5 (32.7-74.9) | 92 (86.7-95.4) | - | 6/9 |
| Zieleskiewicz, L. 2018 (79) | Abdominal | Intraperitoneal effusion | 70 | 96 | - | - | - | 5/9 |
| Scharonow 2018 (75) | Abdominal | Ruling out intraabdominal fluid | - | 97.1 | - | - | - | 5/9 |
| Brun, PM. 2014 (76) | E-fast | Intraperitoneal,  pericardial, and pleural effusion | 95.2 | 95.2 | 95.2 | 95.2 | - | 7/9 |
| Zieleskiewicz, L. 2018 (79) | FAST | Peritoneal effusion | 70 | 96 | 78 | 95 | 92 | 5/9 |
| Scharonow 2018 (75) | Thoracic | Ruling out pneumothorax | - | 100 | - | - | - | 5/9 |
| Donmez, H. 2012 (72) | Thoracic | Correct pneumothorax | 91,4 | 97 | 91,4 | 97 | 97 | 6/9 |
| Ziapour, B. 2015 (73) | Thoracic | Correct pneumothorax | 78 (52-93) | 92 (83-97) | 74 (49-91) | 94 (85-98) | - | 6/9 |
| Press, GM. 2014 (69) | Lung | Pneumothorax | 18.7 (8.9-33.9) | 99.5 (98.2-99.9) | 80 (44.2-96.5) | 92.7 (89.9-94.8) | - | 6/9 |
| Zieleskiewicz, L. 2018 (79) | Lung | Detection of Pneumothorax | 69 | 99 | 94 | 96 | 96 | 5/9 |
| Zieleskiewicz, L. 2018 (79) | Lung | Detection of Haemothorax | 48 | 100 | 90 | 97 | 96 | 5/9 |
| Yates, JG.  2017 (78) | POCUS | Identify pneumothorax, haemothorax and free abdominal fluid | - | - | 100 | 98.3 | - | 6/9 |
| Yamanoglu, A. 2019 (81) | Vena Cava after 500 ml blood loss | Change IVC after exhalation > 1.1mm | 74 | 77 | 79.8 | 70.2 | - | 7/9 |

**Lactate Studies (Quality check):**

| **Study:** | **Outcome:** | **Sensitivity: % (95% CI)** | **Specificity: % (95% CI)** | **Newcastle Ottawa Scale:** |
| --- | --- | --- | --- | --- |
| Lavery, R. 2000 (43) | Lived/Died | 95 (84-99) | 43 (38-48) | 9/9 |
| Guyette, F. 2015 (36) | Resuscitative Care | 93 (84-94) | 48 | 9/9 |
| Guyette, F. 2011 (29) | Emergency Operation | 86 (77-93) | 25 (22-28) | 9/9 |
| Martin Rod, F. 2020 (28) | Mortality | 89.6 (83.2-93.7) | 77.5 (75.9-79) | 8/9 |
| Strnad, M. 2015 (37) | Mortality | 82 (63-94) | 75 (59-87) | 7/9 |

**Intracranial Pressure (Quality check):**

| **Study:** | **Outcome:** | **Sensitivity: % (95% CI)** | **Specificity: % (95% CI)** | **Newcastle Ottawa Scale:** |
| --- | --- | --- | --- | --- |
| Golshani, EZ. 2015 (50) | Raised ICP (CT-signs) | 100 (84-100) | 31.9 (23.0-41.7) | 8/9 |
| Kaur, A. 2021 (59) | Raised ICP (CT-signs) | 93.2 | 91.1 | 9/10 |
| Rajajee, V. 2011 (52) | ICP > 20 mmHg | 96 (91-99) | 94 (92-96) | 8/9 |
| Rajajee, V. 2011 (52) | ICP > 20 mmHg | 98 (89-100) | 91 (88-94) | 8/9 |
| Rajajee, V. 2011 (52) | ICP > 20 mmHg | 100 (85-100) | 92 (83-97) | 8/9 |

**Capnometry (Quality check):**

| **Study:** | **Outcome:** | **Sensitivity: % (95% CI)** | **Specificity: % (95% CI)** | **Newcastle Ottawa Scale:** |
| --- | --- | --- | --- | --- |
| Childress K. 2018 (68) | Mortality | 89 (51-99) | 68 (59-76) | 9/10 |

**Sonography (Quality check):**

| **Study:** | **Outcome:** | **Sensitivity: % (95% CI)** | **Specificity: % (95% CI)** | **Newcastle Ottawa Scale:** |
| --- | --- | --- | --- | --- |
| Brun, PM. 2014 (76) | Intraperitoneal,  pericardial, and pleural effusion | 95.2 | 95.2 | 7/9 |
| Ketelaars, R. 2019 (77) | Hemoperitoneum | 31.3 | 96.7 | 7/9 |

**Lactate Studies (Overview):**

| **Author + Year:** | **Study Design + Patient:** | **Lactate Measurement:** | **Outcome:** | **Cut-off Values:** | **Major Findings:** |
| --- | --- | --- | --- | --- | --- |
| Brown, JB. 2016  (25) | **Design:**   - Cohort study   **Population:**   - 6347 patients - Air rescue after trauma | **Time of measurement:**   - Preclinical (LAC).   **Device:**   - Lactate pro meter (Arkray, Japan)   **Blood collection:**   - Peripheral venepuncture | **Trauma Center Need (TCN):**  **1:** For ACS algorithm  **2:** For ACS + LAC | 1. **None TCN:** LAC <2,6 mmol 2. **TCN :** LAC >3,8 mmol/l | 1. ACS + LAC reduces over-triage by 7.2% and lifts under-triage by 0.7%. 2. ACS+LAC: sensitivity 87.7%; specificity 70%; ROC AUC 0.789. 3. ACS: sensitivity 88.4%; specificity 62.8%; ROC AUC 0.757. 4. ACS+LAC upgraded 256 patients to TCN and downgraded 548 patients from TCN compared to ACS.   **Conclusion:** An addition of LAC to ACS classifies patients into a  more appropriate TCN level. |
| Costa, L.G. 2017  (26) | **Design:**   - Case Control Study   **Population:**   - 200 high energy trauma patients | **Time of measuerment:**   1. prehospital (t1) 2. in emergency department (t2) 3. 3 hours after admission (t3) 4. 24 hours after admission (t4)   **Device:**   - /   **Blood collection:**   - Venepuncture | 1. Mortality within the first 30 days | 1. / | 1. Mean survivors (mmol/l): **T1: 4.47**; t2: 4.42; t3: 3.79; t4: 3.5 vs. mean deceased (mmol/l): **T1: 6.83**; t2: 7.04; t3: 7.01, t4: 5.29. 2. An increase of 1mmol/l within the 4 measurements is associated with a 6% higher probability of death. OR (95% CI: 1.060 (1.029-1.093) P < 0.001 (significant). 3. Lactate levels are independent predictor of early mortality. |
| Fukuma, H. 2019  (27) | **Design:**   - Cohort study   **Population:**  435 trauma patients:  2 cohorts:  1. cohort: 350  2. cohort: 85 | **Time of measurement:**  1. prehospital (LAC Scene)  2. arrival emergency room (LER)  **Device LAC Scene:**   - Lactate Pro, Arkray, Kyoto, Japan   **Device LER:**   - BGA ABL800, Radiometer, Copenhagen, Denmark   **Blood Collection LAC Scene:**   - Peripheral venepuncture   **Blood Collection LER**:   - / | 1. Need intervention for bleeding (surgical/radiological/blood transfusion within 24 h). 2. Need for massive transfusion (>10 units RBCs) 3. Does Lac Scene as a supplement to physiological variables (physiol.) improve the prediction for immediate intervention for bleeding? | 1. Lac Scene 2.8 mmol/l (Cohort 1) 2. LAC Scene 3.1 mmol/l (Cohort 1+2) | 1. Optimal significant cut-off value for need of massive transfusion is 3.1 mmol/l (LAC Scene) (sensitivity 64.7%; specificity 83.5%, ROC AUC 0.764; p < 0.0001). 2. Optimal significant cut-off value for need of intervention for bleeding is 2.8 mmol/l (Lac Scene) (sensitivity 68.2%; specificity 80.1%; ROC AUC 0.764; p < 0.0001). 3. LAC Scene, as an adjunct to physiological variables, improves prediction for immediate intervention for bleeding compared to using physiological variables alone.  - Physiol + LAC Scene: sensitivity 83.3%; specificity 84.2%; ROC AUC 0.882; - Physiol.: sensitivity 79.5%; specificity 76.4%; ROC AUC 0.837 |
| Martin-Rodriguez,F 2020  (28) | **Design:**   - Case Control study   **Population:**   - 2997 patients with acute disease partly trauma | **Time of measurement:**   - Pre-clinical at first patient contact (pLA)     **Device:**   - Epoc Blood Analysis System (Siemens Healtcare GmbH)   **Blood Collection:**   - Venosus | 1. Deceased up to 2 days after hospital arrival subdivided into:  - Total mortality (GES) - Low mortality (GIS) - High mortality (HS) | **(GES):** pLA: 3,9 mmol/l  **(GIS):** pLA: 1,9 mmol/l  **(HS):** pLA: 4 mmol/l | 1. Preclinical lactate measurement improves prediction for mortality detection. Optimal cut-off values:  - (GES): 3.9 mmol/l; sensitivity 89.6%; specificity 77.5%; ROC AUC 0.867; Odd Ratio 29.5; - (GIS): < 1.9 mmol/l; ROC AUC 0.867; - (HS): > 4 mmol/L; ROC AUC 0.867;  1. median pLA:  - Survivors: 2.7 mmol/l - Deceased: 5.7 mmol/L |
| **Author + Year:** | **Study Design + Patient:** | **Lactate Measurement:** | **Outcome:** | **Cut-off Values:** | **Major Findings:** |
| Guyette, F. 2011  (29) | **Design:**   - Cohort study   **Population:**   - 1168 helicopter trauma patients | **Time of measurement:**   - Preclinical at first contact (pLA).   **Device:**   - Lactate Pro, FACT, Canada   **Blood Collection:**   - peripheral venepuncture | 1. Hospital mortality (KM) (no further information) 2. Emergency surgery (in first 24 hours of hospitalization) 3. Multiple organ failure (MODS) | 1. > 2 mmol/l | 1. Addition of pLA > 2mmol/l to further shock criteria (initial shock, respiratory emergency, altered consciousness):  - **Emergency OP**: raises sensitivity from 64% to 86%; lowers specificity from 51% to 25%; PPV from 9% to 8%; NPV from 95% to 96%. - **MODS:** raises sensitivity from 94% to 99%; lowers specificity from 53% to 25%. - **KM:** raises sensitivity from 88% to 97%; lowers specificity from 52% to 25%; PPV from 9.8% to 7%; NPV from 98% to 99%  1. Median pLA deceased significantly higher than survivors (3.8mmol/l vs. 2.3 mmol/l p < 0.0001). 2. pLA measurement significantly improves prediction for mortality, emergency surgery and MODS :  - **KM:** OR (95% CI: 1.23 (1.14-1.34)) - **Emergency OP:** OR (95% CI: 1.33 (1.05-1.21)) - **MODS:** OR (95% CI: 1.14 (1.03-1.23)) |
| Jansen, TC. 2008  (30) | **Design:**   - Cohort study   **Population:**   - 124 patients | **Time of measurement:**   1. prehospital on arrival (t1) 2. arrival at emergency department (t2)   **Device:**   - Accutrend, Roche Diagnostics, Mannheim, Germany.   **Blood Collection:**   - Capillary or venous | **1:** Hospital mortality (no further information) | **(t1):** > 3,5mmol/l  **(t2):** > 3,5 mmol/l | 1. A cut-off value of 3.5 mmol/l or greater at (t1) is the best value to predict death. Sensitivity 75%; specificity 63%, ROC AUC 0.69; PPV 41%; NPV 88%. 2. Mean lactate:  - Deceased (t1): 5.3mmol/l. - Survived (t1): 3.7 mmol/l - Deceased (t2): 5.4 mmol/l - Survived (t2): 3.2 mmol/l  1. A cut-off value of 3.5 mmol/l or greater at (t2) is the best value to predict death. Sensitivity 64%; specificity 74%; ROC AUC 0.72; PPV 47%; NPV 85%. 2. Patients with lactate > 3.5mmol at (t1) and (t2) had higher mortality (41% and 47%) than patients below 3.5mmol/l (12% and 15%). |

| **Author + Year:** | **Study Design + Patient:** | **Lactate Measurement:** | **Outcome:** | **Cut-off Values:** | **Major Findings:** |
| --- | --- | --- | --- | --- | --- |
| Van Beest, PA. 2009  (31) | **Design:**   - Case control study   **Population:**  2 groups: 216   1. non-shock group: 81 2. shock group: 135  - 2a: Subgroup 1: (<4mmol/l) 74 - 2b: Subgroup 2: (>4mmol/l) 61 | **Time of measurement:**   - preclinical (pLA)   **Device:**   - Accutrend, Roche Diagnostics, Mannheim, Germany   **Collection:**   - capillary or venous | **Relationship between pLA and:**   1. Mortality (no further information) 2. ICU stay duration 3. Hospital stay duration | 1. pLA > 3.2 mmol for increased mortality | 1. pLA cut-off value of 3.2 mmol/l is the optimal value to indicate death; sensitivity 75%; specificity 72%; ROC AUC (0.775). 2. Mortality in shock group with pLA > 4 mmol/l is significantly higher than in shock group with pLA < 4 mmol/l (44.3% vs. 12.2%) P < 0.0001 3. Median lactate of shock group was significantly higher than median of non-shock group (3.9 mmol/l vs. 2.8 mmol/l) 4. Hospital mortality was significantly different between shock group and non-shock group (26.7% vs. 1.2%) 5. Significant differences were found between subgroup 1 and subgroup 2 in the length of stay in ICU or hospital. 6. Association between pLA and mortality |
| Del Brio-Ibanez, Pablo 2020 (32) | **Design:**   - Case control study   **Population:**   - 1341 different diseases (trauma incl. 8.6%) | **Time of measurement:**   1. prehospital lactate (pLA) 2. hospital lactate (hLa)   **Device:**   - Accutrend®Plus meter (Roche Diagnostics, Mannheim, Germany   **Blood Collection:**   - Venous blood sample | 1. In-hospital mortality within 48 h from any cause. 2. in-hospital mortality at 7 and 30 days | 1. > 2mmol/l | 1. Median pLA total Population: 3.3 mmol/l (2.2-4.8). 2. Median pLA 2-Day Mortality: 5.5 mmol/L (4.4-7.6): prognostic accuracy of the 2 day mortality of pLA is good, with an AUROC of 0.800 (95% CI: 0.74–0.85; p < 0.001). 3. Median pLA 7 Day Mortality: 4.9mmol/l (3.9-7.0); significant correlation between plA and 7 Day mortality (p<0.001). 4. Median plA 30 Day Mortality: 4.6 mmol/l (3.1-6.9); significant correlation between pLA and 30 Day mortality (p<0.001). 5. Cut off < 2mmol/L -> 2 -Day mortality 0.4 %; Cut Off > 2mmol/l -> 2-Day mortality 9.6 %. Significantly longer in hospital survival at 2 days in patients with lactate < 2mmol/ compared with patients with higher levels p=0.001). |
| Harrois, Anatole 2018  (33) | **Design:**   - Case control study   **Population:**   - 3111 trauma patients | **Time of measurement:**   - Early after admission at hospital   **Device:**   - /   **Blood collection:**   - / | 1. Identification of early hospital risk factors for (acute kidney injury) AKI 2. Early AKI: occurring during first 5 days 3. Late AKI: occurring after 5 days | 1. AKI defined according to the risk, injury, failure, loss of kidney function and end-stage kidney disease (RIFLE) classification from serum creatinine only. | 1. Median Lactate for no early AKI or AKI Stage R: 1.9 mmol/l (1.1-3). 2. Median Lactate for Early AKI Stage I or F: 4.0 mmol/L (2.6-8.). 3. Significant difference between 1. and 2. P >0.001 = early AKI significant higher Median Lactate than late AKI. 4. Blood lactate is independent risk factor for predicting AKI. |
| Kim, Myoung Jun 2021  (34) | **Design:**   - Case-Control study   **Population:**   - 1404 trauma Patients -> after exclusion criteria 148 patients with pelvic ring fracture were enrolled -> divides in Group A (evident arterial bleeding) and Group B (without evidence of pelvic arterial bleeding) | **Time of measurement:**   - Initial laboratory   **Device:**   - /   **Blood Collection:**   - Arterial blood | 1. Laboratory variables (blood lactate) compared between the two groups (A   and B) | 1. > 3.4 mmol/l | 1. Significant groups differences: Group A 60.7% > 3.4mmol/l vs. Group B 26.7% > 3.4mmol/l P= 0.001. 2. A serum lactate > 3.4 mmol/L (OR = 4.589, 95% CI = 1.354–15.552, P =0.014) is revealed as independent risk factors for pelvic arterial bleeding. 3. A serum lactate level > 3.4 mmol/L is an independent risk factor of arterial bleeding in blunt trauma patients with pelvic bone fractures. |
| **Author + Year:** | **Study Design + Patient:** | **Lactate Measurement:** | **Outcome:** | **Cut-off Values:** | **Major Findings:** |
| St. John Alexander E. 2018  (35) | **Design:**   - Cohort study   **Population:**   - 314 normotensive severe trauma patients | **Time of measurement:**   - Prehospital (PLac)   **Device:**   - (Lactate Pro, Arkray Inc., Kyoto, Japan)   **Blood Collection:**   - Intravenous line | 1. PLac’s ability to predict the need for resuscitative care (RC) | 1. > 2.5 mmol/L | 1. The AUROC for prehospital lactate prediction of need for RC was 0.716 (95% confidence interval [CI] [0.632 – 0.800]). 2. Prehospital lactate level of 2.5 mmol/L or greater have a sensitivity of 74.6% and specificity of 53.4% for predicting need for RC. 3. The odds ratio for need for RC associated with a 1-mmol/L increase in PLac is 1.29 (95% confidence interval [CI] [0.40 – 4.12]) for PLac < 2.5 mmol/L; 2.27 (1.10 – 4.68) for PLac from 2.5 to 4.0 mmol/L; and 1.26 (1.05 – 1.50) for PLac ≥ 4 mmol/L. |
| Guyette, Francis 2015  (36) | **Design:**   - Cohort study   **Population**   - 387 blunt or penetrating trauma patients (prehospital SBP between 70 and 100 mmHg) | **Time of measurement:**   - Prehospital Lactate (P-LAC)   **Device:**   - (Lactate Pro, Arkray, Japan)   **Blood collection:**   - following intravenous (IV) line placement | 1. Compare prehospital P-LAC with prehospital hypotension for predicting the need for early RC in trauma patients 2. RC: deﬁned as any of the following within 6 hours of ED arrival:  - blood transfusion of 5 U or greater - intervention for hemorrhage including thoracotomy, laparotomy, pelvic ﬁxation or interventional radiology embolization - death (including death before hospital arrival) | 1. > 2.5 mmol/L | 1. Mean Lactate of Group “need for RC” is 6.2 mmol/L (+-3.7) while Mean Lactate for group “no need for RC” is 3.7 mmol/l (+-3.4). 2. 92.9% of patients in group “Need for RC” have a Mean Lactate of >2.5 mmol/; Difference of 2.5 mmol/l (1.6 -3.5) between booth groups. (no data available if difference is significant) 3. Cut off point of PLAC > 2,5 mmol/ for early resuscitative care has a specificity of 48% and sensitive of 93%. |
| Strnad, Matej 2015  (37) | **Design:**   - Case control study   **Population**   - 70 blunt trauma patients (intubation in prehospital) divided in survivors and non-survivors | **Time of measurement:**   - Arrival in ED   **Device:**   - /   **Blood collection:**   - / | 1. Determine the predictors of in-hospital mortality (Lactate) (no further information) | 1. > 3.4 mmol/L | 1. Optimal Lactate cut-off point for predicting in hospital death: >3.4 mmol/L sensitivity 82%, specifity 95%, PPV 70%, NPV 86%. 2. Significant difference in Median Lactate between Survivors (n=40) and non survivors (n=30): 2.6mmol (2.0-3.5) vs. 5.3 mmol/l (3.7-7.8); P<0.001. |
| Baron, Bonny J. 2018  (38) | **Design:**   - Case control study   **Population:**   - 10.575 Trauma patients - Survived:10.456 - Died: 119 | **Time of measurement:**   - At time of initial evaluation (in ED)   **Device:**   - ABL 800 FLEX Blood Gas Analyzer, Radiometer Medical, Bronshoj, Denmark   **Blood Collection:**   - Venous LAC | 1. In hospital mortality (no further Information) | 1. / | 1. Significant difference in Median Lactate between Survived and Died: 2.3 mmol/L (1.6-3.3) vs. 2.8mmol/L (1.6-4.8); P = 0.0089. |

| **Author + Year:** | **Study Design + Patient:** | **Lactate Measurement:** | **Outcome:** | **Cut-off Values:** | **Major Findings:** |
| --- | --- | --- | --- | --- | --- |
| Brooke, Magdalene 2016  (39) | **Design:**   - Case control study   **Population:**   - 3468 Hemodynamically normal (SBP >90, bpm 60-100) trauma patients | **Time of measurement:**   - At admission in trauma center   **Device:**   - /   **Blood Collection:**   - / | 1. Elevated admission lactate corresponds to the need for massive transfusion in hemodynamically normal trauma patients 2. Massive transfusion (MT) is defined as receiving >10 units of packed red blood cells within 24 hours. | 1. / | 1. Significant difference in Mortality between Patients with Admission Lactate < 4 mmol/l and admission lactate >4mmol/l: 1.9% vs. 7.5%; P<0.001. 2. Significant difference in “Needing MT” between Patients with Admission Lactate < 4mmol/l and admission Lactate > 4mmol/l: 0.3% vs. 2.8%; p<0.001. 3. Patients with (MT) have significant higher initial admission lactate then patients without MT: 5.6 mmol/L vs. 2.6mmolL; p<0.0001. |
| Gale Stephen C; 2016  (40) | **Design:**   - Case control study   **Population:**   - 1829 blunt trauma patients | **Time of measurement:**   - Initial at emergency room   **Device:**   - /   **Blood Collection:**   - / | 1. primary outcome is in-hospital survival 2. secondary outcome for non survivors is early (<24 h) or late (>24 h) mortality | 1. > 4mmol/L | 1. Significant differences in Mean initial lactate between Survivors and Nonsurvivors: 4.20mmol/l (+-2.48) vs. 6.54 mmol/L (+-3.78); P<0.00001. 2. A significant progression in initial lactate values for mortality in study population: early deaths > late deaths > survivors P<0.00001. 3. Mortality is significant higher for patients in the shock group (>4mmol/L) vs. patients without shock (<4mmol/L): 23.5% vs. 8.1% P<0.00001. 4. In shock group signiﬁcant differences persist between early deaths, late deaths, and survivors (P <0.00001). 5. Initial Lactate predicts overall mortality([OR] 1.17; 95% conﬁdence interval [CI]: 1.12-1.23; P <0.00001). |
| Gonzalez-Robledo, J. 2015 (41) | **Design:**   - Before-After study/interrupted time series   **Population:**   - 497 Polytrauma patients | **Time of measurement:**   - At admission in ED   **Device:**   - /   **Blood Collection:**   - / | **1:** To identify factors related to mortality in adult trauma patients. (no further Informations) | 1. > 4mmol/ | 1. One factor for mortality with the strongest association is serum lactate levels > 4mmol/L (OR 9.7) |
| Kaplan, Lewis J 2004  (42) | **Design:**   - Case control study   **Population:**   - 282 Trauma patients requiring vascular repair (torso and extremity) - 64 nonsurvivors - 218 survivors | **Time of measurement:**   - Arrival at ED   **Device:**   - /   **Blood Collection:**   - / | 1. Determines whether acid-base data obtained in the emergency department correlate with outcome from major vascular injury. | 1. / | 1. Significant difference in Mean Lactate between survivors and nonsurvivors: 3.6mmol/l (+-1.5) vs 11.1 mmol/l (+-3.6); p <0.001. 2. All but one nonsurvivor (n=64 ) had initial emergency department lactate > 5mmol/L. |
| Lavery, Robert 2000  (43) | **Design:**   - Case control study   **Population:**   - 375 trauma patients | **Time of measurement:**   - within 10 minutes after admission in trauma center   **Device:**   - /   **Blood Collection:**   - venous (VLAC) | 1. Determine whether VLAC could identify patients with serious injuries | 1. > 2mmol/l | 1. Patients with VLAC >2 mmol/L have significantly increased risk of ISS >13, death, admission to the ICU, and length of stay >2 days. 2. VLAC > 2mmol/l has sensitivity 95%, specificity 43%, PPV 16%, NPV 99% for Lived/Died 3. VLAC > 2mmol/L for admission to the ICU (sensitivity 81%, specificity 51%, PPV 52%, NPV 80%) 4. VLAC >2 mmol/L for emergent operation (sensitivity 70%, specificity 41%, PPV 23%, NPV 85%) |

**Intracranial pressure Studies (Overview):**

| **Author + Year:** | **Study Design + Patient:** | **Brain-pressure Measurement:** | **Outcome + ICP Validation:** | **Cut-off Values:** | **Major Findings:** |
| --- | --- | --- | --- | --- | --- |
| Houze-Cerfon, CH. 2019  (44) | **Design:**   - Case Control study   **Population:**   - 23 patients with moderate and severe traumatic brain injury (TBI) | **Time of measurement:**   - Preclinical   **Device:**   - Ge Vingmed Ultrasound AS, Chalfont St Giles, UK;   **Probe frequency:**   - 7.5 MHz   **Configuration:**   - small areas, 2-dimensional   **Patient position**:   - supine, eyes closed   **Position US:**   - Temporal to eyelid, horizontal   **ONSD:**   - 3mm behind eyeball | **Outcome:**   1. Quality of the US ONSD measurement 2. Feasibility of US ONSD measurement, assessment of user-friendliness 3. Duration US ONSD   **ICP Validation:**   - / | 1. / | 1. Median time to complete is 4 minutes. 2. Median feasibility is 8 out of 10 points. 3. No significant difference between left and right ONSD (5.1mm vs. 5.3 mm) p=0.66. 4. ONSD is significantly higher in patients with severe ICB (median ONSD 5.6mm) vs. patients with moderate ICB (median ONSD 4.8 mm). 5. ONSD US is correctly performed in 71% on scene, 80% in ambulance and 43% in helicopter after validation. 6. Study shows that high quality ONSD measurement with US in prehospital is possible. |
| Geeraerts, T. 2007  (45) | **Design:**   - Case control study   **Population:**   - 31 Patients with TBI: - 16 with normal ICP - 15 with high ICP - 31 control Patients | **Time of measurement:**   - Arrival at ICU, before placement of ICP probe.   **Device:**   - HP Sonos 5500, Hewlett Packard, Les, Ulis, France   **Probe frequency:**   - 7.5 MHz   **Configuration:**   - small areas, 2-dimensional   **Position US:**   - Temporal of eyelid, adjusted to capture ONSD, sagittal and transversal   **ONSD:**   - 3mm behind eyeball; 2 measurements (sagittal and transversal) | **Outcome:**   1. Relationship between US ONSD and ICP in early post-traumatic time window 2. Is an extended ONSD associated with an increase in ICP in the first 48 hours?   **ICP Validation:**   - / | **1:** 5.9 mm | 1. Patients with high ICP (> 20 mmhg over 30 minutes) have significantly greater ONSD than patients with normal ICP ((median) 6.3 mm vs. (median) 5.1mm p< 0.0001). 2. The highest measured ONSD (from both eyes) with a cut-off value of 5.9mm is most accurately to detect increased ICP in the first 48 hours. Sensitivity 87%; specificity 94%; ROC AUC 0.96; PPV 93%; NPV 88%. 3. Significant correlation between ICP and ONSD on arrival. |

| **Author + Year:** | **Study Design + Patient:** | **Brain-pressure Measurement:** | **Outcome + ICP Validation:** | **Cut-off Values:** | **Major Findings:** |
| --- | --- | --- | --- | --- | --- |
| Blaivas, M. 2003  (46) | **Design:**   - Case control study   **Population**:   - 35 patients: 14 with confirmed EICP on CT | **Time of measurement:**   - Emergency room   **Device:**   - Agilent Image Point HX (Phillips, Andover, MA)   **Probe frequency:**   - 10-MHz   **Patient Position:**   - Supine Position   **Position US**:   - on closed eyelid, bilateral, sagittal and transverse   **ONSD:**   - 3mm behind eyeball, bilateral, intersection of both eyes determine ONSD | **Outcome:**   1. US ONSD detects the presence of increased intracranial pressure (EICP)   **ICP Validation:**   - Computed tomography (CT) ﬁndings deﬁned as indicative of EICP: - the presence of mass effect with a midline shift 3 mm or more - a collapsed third ventricle - hydrocephalus - the effacement of sulci with evidence of signiﬁcant edema - abnormal mesencephalic cisterns. | **1:** EICP: ONSD > 5mm | 1. Significant difference between Mean ONSD in patients with and without EICP. (ONSD 6.27mm vs. ONSD 4.42mm) (p=0.001). 2. A cut-off value of ONSD > 5mm has a sensitivity 100%; specificity 95%; PPV 93%; NPV 100%; to detect an increase in EICP (validated with CT). |
| Moretti, R. 2009  (47) | **Design:**   - Case control study   **Population:**   1. Group: 53 patients with ICP probe (primary ICB) 2. Group: 53 patients without ICB | **Time of measurement:**   - Stay in ICU   **Device:**   - Hitachi EUB 405, Hitachi Medical Corporation, Tokyo, Japan   **Probe frequency:**   - 7.5 MHz   **Patient:**   - Supine Position   **Position US:**   - on closed eyelid, bilateral, sagittal and transversal   **ONSD:**   - 3mm behind eyeball, bilateral, ONSD = average of both sides | **Outcome:**   1. Evaluate feasibility of ONSD US 2. Correlation between ONSD and ICP on arrival at ICU 3. Validate Cut-Off of 5mm   **ICP Validation:**   - In 32 Patients ICP monitoring was carried out through an extraventricular drain - in 21 patients through an   intraparenchymal bolt | **1:** ICP (>20mmhg): ONSD > 5.2mm | 1. Group with ICP > 20mmhg has significantly higher mean ONSD than group with low ICP (ONSD 6.2mm vs. ONSD 5mm) (P<0.01). 2. Cut-off value for ICP > 20mmhg: ONSD 5.2mm; sensitivity 94%; specificity 76%; ROC AUC 0.89. |
| Goel, RS. 2008  (48) | **Design:**   - Case control study   **Population:**   - 100 patients | **Time of measurement**:   - in hospital   **Probe frequency:**   - 7.5 MHz   **Patient:**   - supine position   **Position US:**   - on closed eyelid, bilateral, vertical and horizontal   **ONSD:**   - 3mm behind eyeball, bilateral, sagital and transversal, ONSD results from average of both eyes | **Outcome:**   1. Accuracy of ONSD dilation measurement to predict increased ICP   **ICP Validation:**   - CT - Signs for raised ICP in CT: - signiﬁcant oedema, midline shift of 3 mm or more - mass effect - effacement of sulci - collapse of ventricles - compression of cisterns | **1:** ONSD > 5mm: abnormal ICP | 1. Significant difference between mean ONSD in patients with and without ICP: (ONSD with ICP: 5.8mm vs. ONSD without ICP: 3.5mm) (p<0.0001). 2. The cut-off value of ONSD >5mm for signs of increased ICP on CT has a sensitivity of 98.6%, spec of 92.8%, PPV of 97.26%, NPV of 96.3%. |

| **Author + Year:** | **Study Design + Patient:** | **Brain-pressure Measurement:** | **Outcome + ICP Validation:** | **Cut-off Values:** | **Major Findings:** |
| --- | --- | --- | --- | --- | --- |
| Aduayi, Olufunso Simisola 2015 (49) | **Design:**   - Cross-sectional study   **Population:**   - 160 Patients: - 80 with intracranial space occupying lesion (SOL) with possibly raised ICP - 80 without SOL and without clinical suspicion of raised ICP (controls) | **Time of measurement:**   - ONSD measured before cranial CT (in SOL group) - ONSD measured after CT (no raised ICP group)   **Device:**   - Ultrasound unit: Mindray real-time ultrasound scanner model DC-6; Shenzhen, China)   **Probe frequency:**   - 7.5 MHz linear probe   **Patient Position:**   - Supine position, closed eyes closed   **Position US:**   - On temporeal area of eyelid, axial view of the orbit   **ONSD:**   - Retrobulbar 3 mm behind globe. MEAN binocluar ONSD is calculated from the mean ONSD of each eye. | **Outcome:**   1. The use of optic nerve sonography (ONS) for predicting raised ICP in a resource-limited environment.   **ICP Validation:**   - CCT - Signs for raised ICP: - effacement of sulci - midline shift - dilated ventricles - the collapse of ventricles - compression of cisterns. | 1. ONSD > 5.2 mm | 1. Significant difference in SOL group: SOL with raised ICP vs. SOL without raised ICP: Mean ONSD 5.7mm +- 0.59mm vs. 4.8mm +- 0.39mm) P=0.0001). 2. Significant difference between Sol group with raised ICP and Control group without raised ICP: Mean ONSD 5.7mm +- 0.59mm vs. 4.5mm +- 0.22 mm P=0.0001). 3. Cut-off value of MEAN ONSD >5.2mm (sensitivity 81.2%/ specificity 100%) as best predictor for raised ICP. |
| Golshani, Ebrahim Zadeh  2015 (50) | **Design:**   - Case control study   **Population:**   - 131 patients suspected with ICP elevation after different diagnosis (head trauma included) | **Time of measurement:**   - Before CT scan in ED   **Device:**   - **(**HS2000, Honda, Korea)   **Probe frequency:**   - 7.5 mega Hertz   **Patient Position:**   - Eyes in supine position   **Position US:**   - Center of each eye.   **ONSD:**   - Each optic nerve is measured three times and the mean number recorded. - The mean value of two right and left optic nerve sheaths are entered | **Outcome:**   1. To evaluate the diagnostic accuracy of ophthalmoscopy and ultrasonography of optic nerve sheath in compare to brain CT scan in prediction of elevated ICP.   **ICP Validation:**   - CT - Signs for raised ICP: - Cerebral edema - midline structural shift - ventricular collapse - ventricular enlargement - cistern compression | 1. ONSD > 5mm = raised ICP | 1. Ultrasound examination revealed that mean right and left ONSD were 5.5 ± 0.9 (range: 3.8 - 8.7) and 5.5 ± 1.0 (range: 2.0 - 8.6). 2. With cut-off point of 5 millimetres, 102 patients (77.9%) are categorized in the elevated ICP group, based on ONSD. Sensitivity and specificity of ultrasonography in prediction of elevated ICP are 100.0% (95% CI: 84.0 - 100.0) and 31.9% (95% CI: 23.0 41.7). |
| Hanafi, Mohammad Ghasem 2019 (51) | **Design:**   - Case control study   **Population:**   - 112 Patients: - Group 1: 62 traumatic Patients - Group 2: 50 voluntaries healthy | **Time of measurement:**   - in the emergency department   **Device:**   - SONOSCAPE-SSI 6000 (Shenzhen, Guangdong, P.R China)   **Probe frequency:**   - 5-10 MHz   **Patient Position:**   - Supine position, eyes closed   **Position US:**   - A diameter of 3 mm was measured in the posterior to the globe   **ONSD:**   - Three times on each eye in an axial region, the mean of these sizes is obtained as ONSD. | **Outcome**:   1. To assess the efficacy of ONSD sonography in patients with increased ICP induced by trauma.   **ICP Validation:**   - Ct scan - Signs for raised ICP - midline shifts 3 mm or more - third ventricle collapse - hydrocephalus - sulcal effacement with significant edema symptoms | 1. 5.3 mm | 1. Insignificant correlation (r= -0.108, P= 456) of Mean ONSD both sides between trauma Patients vs. Healthy voluntary: 6.06mm +-0.75mm vs. 4.02+-1.07mm. 2. The ONSD in the right and left sides had high and significant correlation in the patients (r = 0.929, P < 0.000) and voluntary healthy (r = 0.630, P < 0.000) group. 3. The critical values of the risk for raised ICP Mean ONSD is 5.3 mm, the sensitivity is 96.4% and the specificity is 71.4%. |
| **Author + Year:** | **Study Design + Patient:** | **Brain-pressure Measurement:** | **Outcome + ICP Validation:** | **Cut-off Values:** | **Major Findings:** |
| Kaur, Amandeep 2021  (59) | **Design:**   - Cross-sectional study   **Population:**   - 100 traumatic brain injury (TBI) with suspected elevated ICP | **Time of measurement:**   - All patients admitted to neurosurgical ICU underwent optic nerve sheath diameter (ONSD) sonography of both eyes followed by CT scan head subsequently.   **Device:**   - /   **Probe frequency:**   - 10-13 MHz linear probe   **Patient Position:**   - Supine position, closed upper eyelid   **Position US:**   - Placed on superior and lateral aspect of the orbit   **ONSD:**   - Transverse ONSD is measured 3 mm behind the retina - Three ONSD measurements on each eye of the patient and the mean of left and right eye ONSD measurements was calculated to minimize interobserver variability | **Outcome:**   1. The bedside sonographic measurement of ONSD can reliably predict elevated ICP in neuro-trauma patients   **ICP- Validation:**   - CT - Signs for raised ICP: - significant edema - midline shift of 3 mm or more - mass effect - effacement of sulci - collapse of ventricles - compression of cisterns | 1. Binocular ONSD > 5.0 mm is considered raised | 1. With a Cut-off of >5.0mm the sensitivity of the bedside sonographic measurement ONSD to detect raised ICP is 93.2% and specificity is 91.1%. Positive Predictive Value is 89.1% and NPV is 94.4%. 2. ONSD measurement is increased in patients with low GCS (3–8) and the relationship between GCS and ONSD readings is highly significant (P-value = 0.000). 3. Out of 46 patients with raised ONSD (≥5.0 mm), 41 patients (89%) have CT findings of raised ICP while 5 patients (11%) did not have finding suggestive of raised ICP. |
| Rajajee, Venkatakrishna, 2011 (52) | **Design:**   - Cross-sectional study   **Population:**   - 65 Patients (different diagnosis, TBI and intracerebral haemorrhage included) | **Time of measurement:**   - Enrolment and intermittently during the course of the patients’ stay in the ICU   **Device:**   - Sonosite TM M-Turbo (SonoSite Inc., Bothell, WA, USA)   **Probe frequency:**   - 13-6 MHz   **Patient Position:**   - /   **Position US:**   - Placed on the superior and lateral aspect of the orbit against the upper eyelid with the eye closed and angled slightly caudally and medially   **ONSD:**   - Three ONSD measurements are attempted on each side for a total of at least 6 attempted measurements per measurement cluster - ONSD is measured 3 mm behind the retina - The mean of all 6 ONSD measurements (3left+3right) is taken | **Outcome:**   1. Validate bedside technique of optic nerve ultrasonography (ON-US) for the detection of intracranial hypertension, using a point-of-care ultrasound machine 2. To identify the optimal ONSD cut-off for the identiﬁcation of ICP.   **ICP Validation:**   - Simultaneous measurement of intracranial pressure via invasive monitoring CT and MR scans of brain within 24 h - Raised ICP > 20 mm/hg | 1. > 4.8 mm | 1. The median ONSD for measurements corresponding to invasive ICP >20 mmHg is 0.53 cm (IQR 0.51–0.57 cm) while the median ONSD with invasive ICP < 20 mmHg is 0.40 cm (IQR 0.36–0.43 cm). difference is statistically significant P< 0.0001). 2. Optimal single ONSD cut-off for the detection of invasive ICP >20 mmHg is > 0.48 cm. The sensitivity of this cut-off is 96% (95% CI 91–99%), speciﬁcity 94% (95% CI 92–96%), positive predictive value 84% (95% CI 77–89%) and NPV 99% (95% CI 97–100%). 3. Optimal single ONSD cut-off for the detection of invasive ICP >25 mmHg is >0.52 cm sensitivity 98% (95% CI 89–100%), speciﬁcity 91% (95% CI 88–94%), PPV 53% (95% CI 42–64%) and NPV 100% (95% CI 99–100%). 4. Optimal mean ONSD cut-off for the detection of mean invasive ICP >20 mmHg is >0.47 cm sensitivity100% (95% CI 85–100%), speciﬁcity 92% (83–97%), PPV 80% (95% CI 61–92%), NPV 100% (95–100%). |

| **Author + Year:** | **Study Design + Patient:** | **Brain-pressure Measurement:** | **Outcome + ICP Validation:** | **Cut-off Values:** | **Major Findings:** |
| --- | --- | --- | --- | --- | --- |
| Robba, Chiara 2020  (53) | **Design:**   - Cross-sectional study   **Population:**   - 100 Patients with traumatic brain injury (TBI), subarachnoid haemorrhage (SAH) or intracerebral haemorrhage (ICH) with invasive ICP monitoring | **Time of measurement:**   - Within the first 72 h after ICP insertion   **Device:**   - Philips iE33, Paris, France   **Probe frequency:**   - 7.5-MHZ l   **Patient Position:**   - Supine position, head elevated to 30 degrees   **Position US:**   - Probe is oriented perpendicularly in the vertical plane and at around 30° in the horizontal plane on the closed eyelids of both eyes without exerting pressure   **ONSD:**   - 3 mm behind the retina in both eyes - Final ONSD value is calculated by averaging four measured values - Abnormal ONSD is considered if > 6.0 mm | **Outcome:**   1. Compare the accuracy of different non-invasive methods to estimate ICP and to estimate the occurrence of intracranial hypertension in a heterogeneous cohort of brain-injured patients.   **ICP Validation:**   - Intraparenchymal fiberoptic transducer, or an extra-ventricular drainage (EVD) catheter inserted into the brain ventricles and connected to an external pressure transducer and drainage system - Intracranial hypertension is defined as ICP > 20 mmHg. | 1. Abnormal ONSD is considered if > 6.0 mm | 1. ONSD > 5.3 mm has 70% sensitivity and 75% specificity to assess intracranial hypertension. 2. There is a significant correlation between the non-invasive technique and invasive ICP: ONSD: r = 0.54. |
| Tayal, Vivek S 2007  (54) | **Design:**   - Cross sectional study   **Population:**   - 59 patients suspected of having elevated intracranial pressure as a result of acute head trauma | **Time of measurement:**   - After presenting to the ED with suspected acute head injury   **Device:**   - Shimadzu (Kyoto, Japan) SDU-400 and SDU 450 gray-scale ultrasonographic machine   **Probe frequency:**   - 7.5 MHz   **Patient Position:**   - Supine position   **Position US:**   - Placed lightly over the closed upper eyelid(s) of the patient   **ONSD:**   - Single optic nerve sheath diameter is measured 3.00 mm behind the globe (Figure 3) in each eye, and then the optic nerve sheath diameter measurements from each eye are averaged to create a binocular optic nerve sheath diameter measurement. - A binocular optic nerve sheath diameter or uniocular measurement in those with one eye measurement (see above) greater than 5.00 mm is considered abnormal | **Outcome:**   1. Does ONSD correlate with increased ICP on CT in an adult patient with traumatic brain injury?   **ICP Validation:**   - CT - Sings for raised ICP: - signiﬁcant edema, midline shift - mass effect - effacement of sulci - collapse of ventricles - compression of cisterns | 1. > 5 mm is abnormal | 1. The mean optic nerve sheath diameter for the 8 patients (ONSD > 5mm) with CT evidence of elevated intracranial pressure is 6.27 mm (95% CI 5.54 to 6.96 mm). 2. The mean binocular optic nerve sheath diameter of the other 51 patients is 4.94 mm (95% CI 4.74 to 5.15 mm), with a difference between groups of 1.33 mm (95% CI 0.766 to 1.866 mm). 3. The sensitivity for the mean binocular optic nerve sheath diameter > 5mm ultrasonography in detecting elevated intracranial pressure is 100% (95% CI 68% to 100%), speciﬁcity 63% (95% CI 50% to 76%). 4. The sensitivity of ultrasonography for detection of any traumatic intracranial injury found by CT is 84% (95% CI 60% to 97%), speciﬁcity is 73% (95% CI 59% to 86%). |

| **Author + Year:** | **Study Design + Patient:** | **Brain-pressure Measurement:** | **Outcome + ICP Validation:** | **Cut-off Values:** | **Major Findings:** |
| --- | --- | --- | --- | --- | --- |
| Cammarata, Gianluca 2011  (55) | **Design:**   - Case control Study   **Population:**   - Case group: 11 Head trauma injured Patients (devides in ICP >20 mmhg and < 20 mmHG) - Controll group: group of 10 ICU patients admitted for nontraumatic Reasons (no invasive ICP monitoring) | **Time of measurement:**   - ONSD ultrasounds is performed within 1 hour of the insertion of ICP probe   **Device:**   - Model Vivid Expert, General Electric Medical System, Milwaukee, WI   **Probe frequency:**   - 10-MHZ   **Patient Position:**   - /   **Position US:**   - Over the temporal area of the eyelid   **ONSD:**   - 3 mm behind the globe - ONSD is measured in both the sagittal and the transverse plane. Each measurement is repeated twice, and values are averaged**.**   **ICP Validation:**   - / | **Outcome:**   1. To determine whether dilation of the optic nerve sheath could reliably identify raises in ICP assessed with an intraparenchymal probe in adult head trauma patients.   **ICP-Validation:**   - Intraparenchymal probe inserted by a neurosurgeon for the measurement of ICP - An ICP <20 mm Hg is considered as normal, whereas values >20 mmHg are classiﬁed as abnormal and treated | 1. / | 1. Head trauma patients without intracranial hypertension (ICP < 20 mmHG) have ONSD values equivalent to those measured in control patients Mean ONSD (5.52 mm +- 0.36 mm vs. 5.51 mm +- 0.32 mm). No significant difference P < 0.0001 2. ONSD values are signiﬁcantly correlated to ICP values (r ⫽0.74, p <0.001) 3. ONSD, instead, signiﬁcantly increased to MEAN ONSD 7.0mm, when ICP rose in value to >20 mm Hg (p <0.0001 vs. normal ICP and control) |
| Girisgin, Abdullah Sadik 2007  (56) | **Design:**   - Case control study   **Population:**   - Group 1: 28 Patients with traumatic or nontraumatic causes with EICP in CT - Group 2: Control 26 healthy volunteers | **Time of measurement:**   - ONSD measurement after EICP finding in CT at emergency apartment   **Device:**   - /   **Probe frequency:**   - 7.5 MHZ   **Patient Position:**   - /   **Position US:**   - /   **ONSD:**   - Vertical and horizontal diameters of the ON in both eyes are measured four times - ONSD is measured 3 mm behind the point where the ON enters the globe | **Outcome:**   1. Evaluate the convenience and utility of optic nerve ultrasonography (ONUS) in the evaluation of emergency patients with elevated intracranial pressure (EICP) due to traumatic or non-traumatic causes   **ICP Validation:**   - EICP determined on cranial computed tomography (CCT) - The findings of EICP in CCT include: - changes in vertical sizes - lessening in basilar cistern sizes - narrowing or eliminating in sulci - transfalcine herniation - changes in the rate of grey/white matter | 1. / | 1. The average of all measurements of the ONSD of 28 patients who had EICP according to CCT is MEAN 6.4 (0.7) mm. The average of all measurements of the ONs of this 26-person control group is 4.6 (0.3) mm. Significant difference between these two groups p<0.001. |

| **Author + Year:** | **Study Design + Patient:** | **Brain-pressure Measurement:** | **Outcome + ICP Validation:** | **Cut-off Values:** | **Major Findings:** |
| --- | --- | --- | --- | --- | --- |
| Major, Robert 2011  (57) | **Design:**   - Case control Study   **Population:**   - 26 Patients (14 trauma, 12 non trauma) | **Time of Measurement:**   - After presenting to emergency department   **Device:**   - /   **Probe frequency:**   - 7,5 MHz   **Patient Position:**   - Closed eyes   **Position US:**   - /   **ONSD:**   - ONSD from both eyes measured and an average taken | **Outcome:**   - To assess if ONSD measurement by ultrasound can accurately predict the presence, or absence, of raised ICP and acute pathology   **ICP-Validation:**   - CT - Sings for raised ICP - Mass effect with midline shift 3 mm or more - Collapsed third ventricle - Hydrocephalus - Effacement of sulci with evidence of signiﬁcant oedema or abnormal mesencephalic cisterns | 1. Positive ONSD measurement is taken as an average ONSD from both eyes > 5 mm. | 1. The sonography ONSD measurement is 100% speciﬁc (95% CI 79% to 100%) and 86% sensitive (95% CI 42% to 99%) for raised ICP, cut off > 5mm. |
| Qayyum, Hasan 2013  (58) | **Design:**   - Cross sectional study   **Population:**   - 24 Patients (13 with TBI) | **Time of Measurement:**   - Admission emergency department   **Device:**   - SonoSite 180 Plus portable ultrasound machine (SonoSite Inc., Bothell, Washington, USA)   **Probe frequency:**   - 7.5 MHz linear   **Patient Position:**   - Closed eyes, supine position   **Position US:**   - /   **ONSD:**   - 3 mm behind the papilla - Two hyperechoic lines behind the globe - Measurement in both eyes | **Outcome:**   - To determine if ultrasound   guided measurement of the ONSD accurately predicted elevated ICP as demonstrated by cranial CT  **ICP-Validation:**   - CCT - Sings of raised ICP: - Shift - Sulcal effacement with significant oedema - Collapse of third ventricle - Hydrocephalus | 1. > 5mm | 1. A Cut-off ONSD > 5mm has a sensitivity of 100%, a specificity of 75%, a PPV of 95.4% and a NPV of 100% for the prediction of raised ICP. |

**Capnometry Studies (overview):**

| **Author + Year:** | **Study Design + Patient:** | **Capnometry Measurement:** | **Outcome:** | **Cut-off Values:** | **Major Findings:** |
| --- | --- | --- | --- | --- | --- |
| Childress, K. 2018  (68) | **Design:**   - Cross-sectional study   **Population:**   - 135 trauma patients | **Time of measurement**:   - Initial at first patient contact   **Device:**   - Microstream capnography with LIFEPEAK 12 devices (PhysioControl, Redmond, WA)   **Measurement:**   - in intubated or non-intubated patients, ETCO2 measured at constant 3-5 breaths | 1. In-hospital mortality 2. Prediction of ETCO2 compared to traditional vital signs | 1. Mortality: EtCO2 < 30 mmHg | 1. Cut off value for in-hospital mortality ETCO2 < 30 mmHg. Sensitivity 89%; specificity 68%; PPV 13%; NPV 99%, ROC AUC 0.84 (p=0.0001). 2. Mean ETCO2 lower in deceased than in survivors (ETCO2 18mmHg vs. ETCO2 34 mmHg) (p< 0.0001). 3. No correlation between blood pressure and ETCO2. |
| Cooper, C J. 2013  (60) | **Design:**   - Case control Study   **Population:**   - 160 Trauma and Burn victims (prehospital intubated) | **Time of measurement:**   - Preclinical   **Device:**   - /   **Measurements:**   - / | 1. ETCO2 does not correlate with PaCO2 2. ETCO2 between 30 and 35 mmHg is associated with avoidable respiratory acidosis | 1. / | 1. Preclinical ETCO2 does not correlate with PaCo2. 2. Preclinical mean ETCO2 was significantly lower than mean PaCO2 on arrival ( 34 mmHG vs. 44 mmHg) (P<0.005). 3. Patients who arrived acidotic were more likely to die. (Mean ph survivors 7.32 vs. Mean pH deceased 7.19). 4. Patients and decedents with pH <7.2 showed the greatest difference between ETCo2 and PaCO2. 5. Conclusion: ETCO2 insufficient to predict PaCO2 and optimise ventilation in severely injured. |
| Deakin, CD. 2004  (61) | **Design:**   - Case control study   **Population:**   - 191 blunt trauma patients | **Time of measurement:**   - Preclinical - (t0): Initial Petco2 after intubation - (t20): Petco2 20 minutes after intubation   **Device:**   - Propq Encore monitor   **Measurement**:   - constant via Sidestream | 1. Relationship between Petco2 and outcome (survival/mortality to discharge) at (t0) and (t20) | **1:** (t0): 3,25 kPA  **2**: (20): 3,25 kPA | 1. Statistically significant difference between (t20) median Petco2 survivors and (t20) median Petco2 deceased (4.10 kPA vs. 3.50 kPA) (p < 0.0001). 2. Petco2 (t20) has better prediction for survival/mortality than Petco2 (0). ROC AUC (t20) 0.76 vs. ROC AUC (t0) 0.62. 3. 5% of patients with Ptco2 < 3.25 kPa survive to discharge. |
| Safari, E. 2020  (62) | **Design:**   - Cohort study   **Population:**   - 250 intubated multiple trauma patients | **Time of measurement**:   - Preclinical - (t0): Initial Petco2 after intubation - (t20): Petco2 after 20 minutes   **Device:**   - IRMA CO2 sample   **Measurement:**   - Via Sidestream | 1. Relationship between ETCO2 and lactate 2. ETCO2 and lactate: predicting hospital mortality (no further information) | 1. / | 1. Inverse relationship between serum lactate and ETCO2 demonstrated (p<0.0001). 2. MEAN Etco2 Deceased vs. Survived ( 19.59 mmHg vs. 36.16 mmHg). 3. ETCO2 shows a significant relationship with hospital mortality ROC AUC 0.97; (p=0.023). |

| **Author + Year:** | **Study Design + Patient:** | **Capnometry Measurement:** | **Outcome:** | **Cut-off Values:** | **Major Findings:** |
| --- | --- | --- | --- | --- | --- |
| Caputo, ND. 2012  (63) | **Design:**   - Cohort study   **Population:**   - 105 penetrating trauma patients | **Time of measurement:**   - On arrival of the trauma team in the emergency department   **Device:**   - Phillips Smart Capnoline Plus, M2526A   **Measurement:**   - nasal cannula via sidestream detector | 1. Correlates ETCO2 with serum lactate, tissue hypoxia and subsequent shock. 2. Can probability of surgical intervention be determined by ETCo2 | 1. / | 1. Strong inverse correlation between serum lactate level and ET CO2 , R = -0.86; (p = 0.0001). 2. 81% surgical patients had a significantly lower ETCO2 than non-surgical patients (Mean ETCo2 surgical 25.2mmHG vs. Mean EtCo2 non-surgical 29.4mmHG p= 0.006). 3. 97% of patients requiring massive transfusion had an ETCO2 < 35mmHg. 4. ODDS ratio for patients with very low ETCO2 (<35mmhg) to receive surgical intervention was 20.4. |
| Takano, Y. 2003  (64) | **Design:**   - Cross-sectional study   **Population:**   - 41 spontaneously breathing patients in a medical ward | **Time of measurement:**   - In hospital   **Device ETCO2:**   - NPB-75 handheld capnograph/pulse oximeter, Nellcor Puritan Bennett Inc., Pleasanton, CA, U.S.A)   **Measurement:**   - Via sidestream - **PaCO2**: arterial CO2 Pressure - **ETCO2:** End tidal CO2 - **TV-ETCO2**: Tide Volumen ETCO2 - **VC-ETCO2**: Vital Capacity VC-ETCO2 - **FEV1.0%:** ratio of forces exspiration volume in 1 sec | 1. Correlation between PaCO2 and ETCO2 in spontaneously breathing patients with or without chronic lung disease. 2. Determines whether ETCO2 measured with TV-Etco2 or with VC-ETCO2 has a more significant correlation with Pa CO2. | 1. / | 1. Mean difference between PaCO2 and VC-ETCO2 was not statistically significant (P= 0.6) VC-ETCO2 shows significant correlation with PaCO2. 2. In summary: VC-ETCO2 measures more accurately than TV-ETCO2 compared with PaCO2 in spontaneously breathing patients with and without lung disease. 3. Significant correlation between VC-ETCO2 and PaCO2 in patients with FEV1.0% below 70% (patients with impaired lung function) (P< 0.0001). 4. Mean difference between PaCO2 and TV-ETCO2 was statistically significant (p<0.0001). |
| Day, Darcy L. 2020  (65) | **Design:**   - Case control study   **Population:**   - 262 Blunt trauma patients assign to the lower-tier modified trauma activation criteria | **Time of measurement:**   - During the initial evaluation in the trauma bay in a 10-minute period   **Device:**   - Oridion Microstream Smart Capnoline Plus nasal sampling set (Covidien/Medtronic, Dublin, Ireland)   **Measurment:**   - EtCO2 nasal cannula (NC) in non-intubated patients in sidestream | 1. EtCO2 values for patients triaged to lower-tier trauma activation are associated with mortality and with the need for critical resources (defined as intubation, blood transfusion, vasoactive medication infusion, surgery/angiography, or ICU admission.) | 1. < EtCO2 30 mmHg | 1. Mean EtCO2 value for patients who required blood transfusion versus not requiring blood transfusion is 26 mmHg vs. 30 mmHg, p = .03 (significant difference). 2. EtCO2 values less than 30 mmHg are significantly associated with blood transfusion (p = .03) but not with other critical resources or mortality. |
| Stone, Melvin E 2017  (66) | **Design:**   - Case control study   **Population:**   - 67 patients requiring the highest level of trauma team activation | **Time of measurments:**   - During primary survey upon patient arrival to ED trauma bay   **Device:**   - /   **Measurments:**   - ETCO2 taken from capnography waveform allowing 5-10 secounds steady waveform - Non intubated: via nasal cannula with side strema detector/ Emergent intubation: via endotracheal tube in line detector | 1. To determine if ET CO2 is independently predictive of the need for MT activation as defined by positive CAT status (CAT+) (CAT+=three units or more of PRBC’s are transfused in a one-hour interval) 2. that abnormally low ET CO2 is associated with more standardized markers for shock, e.g., SBP and lactate | 1. Patients Who Met Traditional Massive Transfusion Criteria (T-MT+) 2. Patients Who Did Not Meet Criteria (T-MT-) | 1. Significant difference in Mean End Tidal CO2 between Died (n=6) and Survivor (n=61): 23.8 mmHG (5.1) vs. 34.6 mmHG (8.2); p =0.002. 2. Significant difference in Mean End tidal CO2 between Shock Index > 0.9 (n=17) and Shock Index < 0.9 (n=50): 29.9 mmHG (7.s) vs. 34.9 mmHG (8.6) p= 0.037. 3. Significant difference in Mean End tidal CO2 between Lactate > 4 mmol/L (n=22) and Lactate < 4 mmol/L (n=45): 29.4 mmHG (8.1) vs. 35.7 mmHG (8.0); p=0.003. 4. Significant difference in Mean ETCO2 between (T-MT+) (n=8) and (T-MT-) (n=59): 27.5 mmHG (8.4) vs. 35.5 mmHG (8.2) p=0.028. 5. Significant difference in Mean ETCO2 between CAT (+) (n=21) and CAT (-) (n=46): 29.5 mmHG (7.5) vs. 35.5 mmHG (8.3) p= 0.006. |
| **Author + Year:** | **Study Design + Patient:** | **Capnometry Measurement:** | **Outcome:** | **Cut-off Values:** | **Major Findings:** |
| Hunter, Christopher L.  2014  (67) | **Design:**   - Case control Study   **Population:**   - 1088 trauma (10%) and medical patients: - 1048 survivors - 40 Non survivors | **Time of measurements:**   - Prehospital by first arriving of EMS   **Device:**   - LIFEPAK 12 devices (PhysioControl, Redmond, WA)   **Measurements:**   - Via microstream capnography when capnographic wave peaks were at a constant end-tidal for 3 to 5 respirations | 1. Death at any point during hospitalization. | 1. / | 1. Significant difference in Mean ETCO2 between Survivors and Non-survivors: (34mmHg (95% CI 34-35 mmHg) vs. 25 mmHG (95% CI 21-29 mmHg ); P< 0.001. 2. Abnormal ETCO2 (<31mmHg or > 41mmHG) for predicting mortality has sensitivity 93% (95%CI 92%-100%), specifity 44% (95% CI 41%-48%), npv 99% (95% CI 92%-100%). |

**Sonography Studies (Overview):**

| **Author + Year:** | **Study Design + Patient:** | **Sonography Measurement:** | **Outcome + Validation:** | **Major Findings:** |
| --- | --- | --- | --- | --- |
| Press, GM. 2014  (69) | **Design:**   - Before-After study   **Population:**   - 293 patients | **Time of measurement:**   - Preclinical during transport   **Device:**   - Portable US (M-Turbo and P-21x transducer; Sonosite)   **Sections (6):**  1. hepatorenal; 2. splenoral; 3. suprapubic;  4. cardiaac (subcostal or parasternal long axis); 5. right lung;  6. left lung  **Interpretation:**   - Lung: pneumothorax exclusion if lung slide present. - Abdomen: exclusion of intraperitoneal fluid - Heart: pericardial fluid exclusion | **Outcome:**   1. Accuracy application of EFAST by emergency medical services (HEMS) in air rescue   **Validation:**   - CT | 1. Diagnosis haemoperitoneum: sensitivity 46%; specificity 94.1% 2. Diagnosis of pneumothorax: Sensitivity 18.7%; Specificity 99.5%. 3. HEMS perform EFAST with moderate accuracy. |
| Walcher, F. 2002  (70) | **Design:**   - Before-After study   **Population:**   - 61 patients with suspected intra-abdominal injury | **Time of measurement:**   - Preclinical   **Device:**   - PRIMEDIC Handy Scan (Metrax GmbH, Rottweil, Germany)   **Probe frequency**:   - 3-5 MHz   **Sections:**  6 section planes:   1. lateral diaphragmatic longitudinal section right 2. lateral caudal longitudinal section right 3. lateral diaphragmatic longitudinal section left 4. lateral caudal longitudinal section left 5. median lower abdomen t.s./l.s. 6. epigastrium, cardially directed | **Outcome:**   1. Detection of abdominal haemorrhage by pre-hospital trauma surgery 2. Influence of investigation on further management of patient care   **Validation:**   - CT | 1. Examination time of 2.8 minutes on average. 2. US findings for detection of intraperitoneal fluid volume validated with CT): specificity 97.5%, sensitivity 100%, positive predictive value 94.2%, negative predictive value 100%. 3. Modification of preclinical management in 36%. 4. 21% influenced choice of destination hospital. 5. 80% of emergency physicians were positive about US. |
| Walcher, F. 2006  (71) | **Design:**   - Before-After study   **Population:**   - 202 patients with suspected abdominal trauma | **Time of measurement:**   - Preclinical   **Device:**   - PRIMEDIC Handy Scan (Metrax GmbH, Rottweil, Germany)   **Probe frequency**:   - 3-5 MHz   **Sections:**  6 section planes P-FAST:   1. lateral diaphragmatic longitudinal section right 2. lateral caudal longitudinal section right 3. lateral diaphragmatic longitudinal section left 4. lateral caudal longitudinal section left 5. median lower abdomen t.s./l.s. 6. epigastrium, cardially directed | **Outcome:**   1. Evaluation of the P-Fast algorithm at the accident site   **Validation:**   - Validation of P-Fast findings with CT and sonography in hospital | 1. Mean examination time 2 -4 min. 2. PFAST for detection of free abdominal blood: Sensitivity 93%; Specificity 99%; Accuracy 99% respectively. 3. 30% adjustment of preclinical management based on P-Fast results. 4. 22% change target hospital after P-Fast findings. 5. 95% of P-Fast implementation was possible without restricting other areas of prehospital care (survey of rescue team). 6. On average P-FAST was performed 35 minutes earlier than CT or sonography in hospital. |

| **Author + Year:** | **Study Design + Patient:** | **Sonography Measurement:** | **Outcome + Validation:** | **Major Findings:** |
| --- | --- | --- | --- | --- |
| Montoya, J. 2016  (83) | **Design:**   - Synopsis and review   **Population:** | / | **Outcome:**   1. Synopsis of focused assessment with sonography for trauma (FAST and E-FAST 2. Review of key pitfalls, controversies, limitations and advances related to FAST, E-FAST and ultrasound education   **Validation:**   - / | 1. Ultrasound can easily detect as little as 200ml of fluid in Morrison`s pouch. 2. The traditional FAST paradigm includes four basic sonographic views (a) pericardial; (b) perihepatic; (c) perisplenic; and (d) pelvic. 3. There are limitations for FAST like poor accuracy in the early post-injury phase, the utility in detecting retropertioneal blood, is unable to identify injuries which aren’t associated with hemoperitoneum. 4. The Combination of the visible „lung sliding effect“ and the „comet tail“ artefact have a 100% negative predictive value for pneumothorax. |
| Donmez, H. 2012  (72) | **Design:**   - Before-After study   **Population:**   - 68 patients included after multiple trauma | **Time of measurement:**   - Arrival emergency room   **Patient position**:   - Spine position   **Device**:   - Nemio scanner (Thosiba, Tokyo, Japan) Probe frequency: 5-Mhz   **Pleural sound:**   - Middle axillary line between fourth and eighth ribs - Middle clavicular line between second and fifth ribs   **Chest in 3 regions:**   1. medial to mid clavicular line 2. between mid clavicular and mid axillary line 3. posterior to the mid axillary line   **Exclusion of pneumothorax:**   - Presence of comet tail artefacts and lung sliding   **Inclusion pneumothorax:**   - Absence of both | **Outcome:**  **1:** Detection of pneumothorax by bedside ultrasound  **Validation:**   - CT | 1. Ultrasound detection of pneumothorax: sensitivity: 91.4%; specificity: 97%; ppV 91.4%; npv 97%; overall accuracy 97%. 2. Loss of sliding lung and comet-tail artefact has high sensitivity and specificity for detection of pneumothorax with ultrasound. 3. Ultrasound more accurate than CXr for detection of pneumothorax. |

| **Author + Year:** | **Study Design + Patient:** | **Sonography Measurement:** | **Outcome + Validation:** | **Major Findings:** |
| --- | --- | --- | --- | --- |
| Ziapour, B. 2015  (73) | **Design:**   - Case control study   **Population:**   - 45 patients with severe multiple trauma | **Time of measurement**:   - After triage in emergency room   **Device:**   - /   **Probe frequency US:**   - 9 MHz   **Patient position:**   - Spine position   **Anterior Convergent:**   - 4 positions on each anterior hemithorax. Lines create a rallying point in the 3rd intercostal space at the level of the middle clavicle line.   **Inclusion of pneumothorax:**   - Pleural line, no sliding sign, no comet-tail artefact | **Outcome**:   1. Verify performance and time efficiency of the Anterior Convergent algorithm   **Validation:**   - CT | 1. US: Mean time 64 sec.. 2. Detection of pneumothorax by US: Sensitivity 78%; Specificity 92%; PPV 74%; NPV: 94%. 3. Anterior Convergent chest US is a suitable method for diagnosis in the first minutes in trauma patients. 4. Sign: "Gestalt Lung Recession" may improve the specificity for detecting pneumothorax. |
| Kirkpatrick, AW. 2004 (74) | **Design:**   - Case Control study   **Population:**   - 225 patients (207 blunt trauma, 18 penetrating trauma) 17 excluded | **Time of measurement**:   - Initial resuscitation Patients who do not require immediate invasise interventions   **Device:**   - Sonosite 180, Sonosite Corporation, Bothell, WA   **Probe frequency:**   - 10.0 – 5.0 MHz (L38 broadband linear array)   **Patient Position:**   - /   **Chest scan:**  Longitudinall on the chest, peripendicular to the ribs ( identifying the pleural interface), thereafter rotation to a tarnsversely position between ribs (identyfing the echogenic pleural stripe)  Evaluation of anteromedial chest at 2nd intercostal space in mid-clavicular line and of the anterolateral chest at 4th or 5th intercostal space in mid axillary line  **Inclusion of pneumothorax:**   - The absence of the „comet-tail“-artefact and the „lung sliding“ | **Outcome:**   1. E-FAST examination for pneumothoraces were compared to subsequent results of various imaging techniques (Chest radiography, CT, etc.)   **Validation:**   - Chest radiography, CT | 1. Sonography and Chest radiography agreed in 89.4 % (186 Patients) regarding the presence or absence of PTXs, in 6 cases (2.9%)CXR was better than eFAST, in 16 cases (7.7%) eFAST was better than CXR. 2. Compared with the composite standard (CXR, chest and abdominal CT scans, clinical course, and invasive interventions), the sensitivity of EFAST was 58.9%, specificity of 99.1% and a likelihood ratio of 69.7. 3. Comparing EFAST directly to CXR, by looking at each of 266 lung fields with the benefit of the CT gold standard, the EFAST showed higher sensitivity over CXR (48.8% versus 20.9%). 4. Both exams had a very high specificity (99.6% and 98.7%), and very predictive LRⴙ(46.7 and 36.3). |
| Scharonow 2018  (75) | **Design:**   - Case control study   **Population:**   - 546 Patients with 99 emergency ultrasounds (31 trauma surgery cases, 68 medical cases) | **Time of measurement:**   - Prehospital setting by rescue physicians   **Device:**   - (Sonosite, MicroMaxx / sector array transducer P17)   **Probe frequency:**   - 5-1 MHz   **Position Patient:**   - /   **Measurement:**  **Three different Ultrasound protocols:**   1. FAST (Focused Assessment with Sonography for Trauma 2. PLUS (Prehospital Lung Ultrasound) 3. FEEL (Focused Echocardiography in Emergency Life support) | **Outcome:**   1. Prehospital ultrasound   findings and diagnoses were compared with the  in-hospital findings obtained in the receiving hospitals:   - left ventricular contractile function, lung interstitial syndrome, ruling out intraabdominal fluid, ruling out pneumothorax, and right ventricular stress.   **Validation:**   - verified at the receiving hospital using ultrasonography, CT scan or x-rays | 1. The emergency ultrasound findings impaired left ventricular contractile function (sensitivity 89.4%, specificity 73,33%; ppv 0,809; npv 0,846); right ventricular stress ( sensitivity 85.7%); lung interstitial syndrome (sensitivity 100%, specificity 96,15%, ppV 1,0, npv 0,96); ruling out pneumothorax (specificity 100%); ruling out intraabdominal fluid (specificity97,1%) in Validation with in hospital findings. 2. The prehospital diagnosis was confirmed in 90.8% of cases, there is no significant difference between prehospital and in-hospital findings ( p-values from p=0.688 to p=0.99). 3. Ultrasound-related changes in patient management occurred in 49.5% of patients; in 33.3% these were transported-related. |
| **Author + Year:** | **Study Design + Patient:** | **Sonography Measurement:** | **Outcome + Validation:** | **Major Findings:** |
| Brun, Pierre-Marie 2014  (76) | **Design:**   - Cohort study   **Population:**   - 98 severe trauma patients | **Time of measurement:**   1. Group 1: Prehospital on site 2. Group 2: Prehospital during transfer 3. Group 3: Both (on site & during transfer)   **Device:**   - TITAN (SONOSITE) with a convex probe   **Probe frequency:**   - 2-4 MHz   **Position Patient**:   - /   **Measurement:**   - EFAST: assesses investigation of intraperitoneal, pericardial and pleural effusion | **Outcome:**   1. Intraperitoneal, pericardial, or pleural effusion or not. 2. The ultrasonography examination is considered positive if it shows pleural, pericardial or peritoneal effusion and negative if no pathologic image was seen   **Validation:**   - The contribution of the prehospital eFAST examination is compared with the results obtained on hospital admission by a radiologist | 1. The eFAST feasibility is 95.4% (Group 1) , 93.9% (Group 2), and 95.2% (Group 3), respectively. 2. The eFAST efﬁciency is 95% (Group1: Sensitivity 95.2%; Specificity 95.2%;PPV 95.2%; NPV 95.2%); 97% (Group 2: Sensitivity 94.7%; Specificity 100%; PPV 100%; NPV 92.3%);, and 100% (Group 3: Sensitivity 100%; Specificity 100%; PPV 100%; NPV 100%); respectively. 3. There is no signiﬁcant difference in performance or duration whether the examination is performed on-site, during transfer or both (w=0.68). 4. Mean and Median On-site eFAST Duration: 3.5 min.; 4 min. 5. Mean and Median Transfer eFAST Duration: 3.9 min.; 4.5 min. 6. Mean and Median in both eFAST Duration: 13 min.; 15min. |
| Ketelaars Rein 2019  (77) | **Design:**   - Before-After study   **Population:**   - 1495 Patients with trauma mechanism or illness | **Time of measurement:**   - Prehospital by Helicopter Emergency Medical Service (HEMS)   **Device:**   1. MicroMaxx (FUJIFILM SonoSite Inc., Bothell, Washington, USA) 2. NanoMaxx (FUJIFILM SonoSite Inc., Bothell, Washington, USA) 3. M-Turbo (FUJIFILM SonoSite Inc., Bothell, Washington, USA)   **Probe frequency:**   - 5-1 MHz roadband phased array transducer   **Position Patient:**   - /   **Measurement:**   - PHUS: abdominal prehospital ultrasound | **Outcome:**   1. To determine the impact of abdominal PHUS on patient care in our region.   **Validation:**   - PHUS diagnostic performance was compared with computed tomography scan or laparotomy | 1. The sensitivity of prehospital abdominal US for hemoperitoneum is 31.3%, specificity is 96.7%, and accuracy is 82.1%. 2. 188 out of 1495 Patients have impacts on treatment after PHUS examination (12.6%). 3. The four main categories of treatment decision impacted by PHUS are information provided to the destination hospital (45.4%); mode of transportation (23.5%); choice of destination hospital (13.1%); and fluid management (11.6%). |
| Yates, Jeffrey G 2017  (78) | **Design:**   - Case-control study   **Population:**   - 190 traumatically injured patients | **Time of measurement:**   - Prehospital in helicopter   **Device:**   - Sonosite M-turbo portable US device (Bothell, WA, USA)   **Probe frequency**:   - /   **Position Patient:**   - /   **Measurement:**   - POCUS in eFAST : - Evaluate the presence of a signiﬁcant amount of “free ﬂuid” in the dependent areas of the abdomen and pelvis, observe for the presence of free pericardial ﬂuid along with a gross evaluation of cardiac activity and function, and l interrogate the thorax for the presence of pleural (lung) sliding in the evaluation for PTX and HTX. | **Outcome:**   1. Identiﬁcation of pneumothorax, hemothorax, and free abdominal ﬂuid in POCUS validated with findings obtained by the trauma team upon initial evaluation at a level 1 trauma center and with the subsequent CT scans that were performed or the surgeon's operative note   **Validation:**   - Obtained by the trauma team upon initial evaluation at a level 1 trauma center and with the subsequent CT scans that were performed or the surgeon's operative note. | 1. Flight Crew POCUS examinations obtained a Positive Predictive Value (PPV) of 100% and a Negative Predictive Value (NPV) of 98.3% for the identiﬁcation of pneumothorax, hemothorax, and free abdominal ﬂuid, which is equivalent to that of the Trauma Team's POCUS studies on the same group of patients. No significant difference between Pocus perfomed by both groups; P= 0.35. |

| **Author + Year:** | **Study Design + Patient:** | **Sonography Measurement:** | **Outcome + Validation:** | **Major Findings:** |
| --- | --- | --- | --- | --- |
| Zieleskiewicz, Laurent 2018  (79) | **Design:**   - Cohort study   **Population:**   - 756 Severe trauma patients | **Time of measurement:**   - Admission in trauma bay   **Device:**   - /   **Probe frequency:**   - /   **Position Patient:**   - /   **Measurement:**   - E-FAST: ultrasonographic assessment of the abdomen, pelvis, pericardium, and pleura - LUS (lung ultrasonography) | **Outcome:**   1. To evaluate the diagnostic accuracy and therapeutic impact of an initial imaging work-up including a CXR, PXR, and eFAST in severe trauma patients   **Validation:**   - With the WBCT (whole-body computed tomography) scan or surgical ﬁndings | 1. Sensitivity and speciﬁcity of the abdominal ultrasound to detect intraperitoneal effusion were 70% and 96%, respectively. 2. The detection of Pneumothorax with LUS has Sensitivity 69%, Specificity 99%; PPV 94%; NPV 96%; Diagnostic accuracy 96%. 3. The detection of Haemothorax with LUS has Sensitivity 48%, Specificity 100%; PPV 90%; NPV 97%; Diagnostic accuracy 96%. 4. The detection of Peritoneal effusion with FAST has Sensitivity 70%, Specificity 96%; PPV 78%; NPV 95%; Diagnostic accuracy 92%. |

| **Author + Year:** | **Study Design + Patient:** | **Sonography Measurement:** | **Outcome:** | **Cut-off Values:** | **Major Findings:** |
| --- | --- | --- | --- | --- | --- |
| Lyon, M. 2005  (80) | **Design:**   - Before-After study   **Population:**   - 31 blood donors | **Time of measurement:**   - When donating blood, directly before and after donation   **Position:**   - Back Position spine   **Device:**   - Sonosite 180 plus portable ultrasound (Sonosite, Bothell, Wash)   **Probe frequency:**   - 2-4 MHz (abdominal setting) with Doppler   **Section:**   - Subxyphoid sagital section of IVC posterior to the liver. - IVCe and IVCi were measured 2 cm distal to the IVC-liver vein inflow. | 1. Changes in IVCd when 450 ml of blood is drawn. 2. IVCd: inferior vena cava diameter 3. IVCi: on inspiration 4. IVCe: on exhalation 5. dIVCe: after blood donation 6. diVCi: after blood donation | **1**: 450ml Blood Collection | 1. Significant difference between IVCe before and after donation was Mean 5.5mm (P< .0001). 2. Significant difference between IVCi before and after donation was Mean 5.16mm (95% CI 4.2-5.9 mm) P < .0001. 3. dIVCe and diVCI correlate closely (r=0.83). 4. Both IVCi and IVCe have an average reduction of 5mm after a blood loss of 450mm. 5. IVC - diameter is a good predictor of blood loss. |
| Yamanoglu, A. 2019 (81) | **Design:**   - Cohort study   **Population:**   - 118 blood donors - 95 control group | **Time of measurement**:   - Before and 15 minutes after blood donation (30 minutes between US)   **Device:**   - Mindray DC-7 (Medical international Limited, Shenzhen, China)   **Probe frequency:**   - 3.5-5 MHz   **Position:**   - Spine position   **Section:**   - Hepatic window in subxiphoid region. - IVC in transverse section with Doppler. Then rotation for longitudinal section - Recording over 3 respiratory cycles | 1. 500ml blood loss clinical outcome in changes of IVC Diameter 2. IVCmax: End expiratory maximum diameter + 3. IVCmin: End-inspiratory minimum diameter | 1. 500ml blood draw | 1. Significant difference between baseline IVCmax and post-IVCmax (p<0.001). 2. Significant difference between baseline IVCmin and post-IVCmin (p<0.001). 3. No significant difference between baseline and post-IVC both min and max in control group. 4. A change in IVCmax > 1.1 mm detects prior haemorrhage with a sensitivity of 74%; specificity of 77%; PPV of 79.8% and NPV of 70.2% (ROC AUC 0.79). 5. IVCmax is a better indicator of bleeding detection than IVCmin. 6. Mean IVEmax decreased by 2.2mm after blood sampling. 7. Patients showed a significant difference in IVC after blood collection compared to before blood collection (IVCmax Mean 17.4mm vs. 15.1mm P<0.001) (IVCmin Mean 10.1mm vs. 8.4 mm P<0.001 |

| **Author + Year:** | **Study Design + Patient:** | **Sonography Measurement:** | **Outcome:** | **Cut-off Values:** | **Major Findings:** |
| --- | --- | --- | --- | --- | --- |
| Patil, S. 2016  (82) | **Design:**   - Before-After Study   **Population:**   - 4126 healthy patients | **Time of measurement:**   - In hospital, no acute setting necessary   **Device:**   - HD7xe, Koninklijke Philips N.V., Amsterdam, The Netherlands   **Probe frequency**:   - 2-4 MHz   **Position:**   - Spine position   **Incision:**   - 1-2 cm right of midline just below the xiphoid. Transverse IVC section shown at intersection of right atrium/liver vein/IVC; longitudinal IVC section same position after rotation; taken during inspiration and expiration. | 1. Determination of the IVC diameter in inhalation and exhalation in healthy patients with subsequent allocation to individual groups according to age, height, weight, BMI. | 1. / | 1. During inspiration, the IVC was between 0.46cm and 1.54cm, the mean is 1.04 cm. 2. During expiration, IVC was between 0.97cm and 2.26cm, the mean is 1.69 cm. 3. During inspiration, IVC decreases in all individuals. 4. During expiration, IVC increases in all individuals. 5. IVC diameter correlates particularly strongly with height, weight and BMI in both men and women. |
